# Distinct cMET inhibitors uncover pharmacological heterogeneity in SHH medulloblastoma cell lines

**DOI:** 10.1007/s12672-026-04717-7

**Published:** 2026-02-23

**Authors:** Sonia Morlando, Alexander W. I. Cox, Aabha Mahesh Thite, Ruth Aidoo, James Wilkinson, Caroline H. Topham, Gianpiero Di Leva

**Affiliations:** 1 Biomedical Research Centre, School of Science, Engineering and Environment, Salford, Greater Manchester, UK; 2 School of Life Sciences, Guy Hilton Research Centre, Keele University, Stoke-on- Trent, UK

## Abstract

The c-MET kinase signalling pathway is crucial in cerebellum development and has emerged as a potential therapeutic target for medulloblastoma treatment. Our study confirms that c-MET and its ligand HGF are highly expressed in the SHH medulloblastoma subgroup, with SHH-α and SHH-β subtypes showing the highest expression levels of both genes, respectively. HGF treatments and siRNA-mediated c-MET knockdown demonstrated the proliferative dependence of SHH MB cells on c-MET signalling. However, time-lapse microscopy revealed heterogeneous behaviours of SHH cell lines following alterations in c-MET levels. Pharmacological inhibition of c-MET signalling showed that tivantinib outperforms other c-MET inhibitors, including crizotinib and foretinib, in killing SHH medulloblastoma cells. Tivantinib induces a prolonged mitotic block followed by apoptotic death in both 2D and 3D cell culture models. Additionally, tivantinib enhances the anti-proliferative activity of vincristine, the standard of care for MB patients, specifically in DAOY cells, where the combination of vincristine and tivantinib synergistically increases the level of PARP cleavage. Moreover, we demonstrated that foretinib and crizotinib induce mitotic slippage in SHH MB cells. The use of navitoclax, a pan-BCL2 inhibitor, initiates the apoptotic program after cells fail mitosis. Taken together, these data suggest that tivantinib is a superior clinical option for SHH MB patients, whereas foretinib or crizotinib should be considered only in combination with BCL2 inhibitors.

**Supplementary Information:**

The online version contains supplementary material available at 10.1007/s12672-026-04717-7.

## Introduction

Medulloblastoma (MB) is one of the most common paediatric brain tumours, originating in the posterior fossa of the cerebellum and often spreading to other parts of the Central Nervous System (CNS) [1]. According to the 2021 World Health Organization (WHO) classification of CNS tumours, MB includes four genetically distinct subgroups: Wingless (WNT), Sonic Hedgehog (SHH, with TP53-wildtype and TP53-mutant subtypes), Group 3 (G3), and Group 4 (G4) [2]. Each subgroup has unique molecular characteristics and clinical outcomes. Recent analyses have further divided MB into multiple subtypes within each subgroup, indicating significant intra- and inter-tumour heterogeneity with potential clinical implications [2]. Despite this heterogeneity, most MB patients receive a uniform treatment regimen consisting of surgery, radiotherapy, and chemotherapy, which often leads to severe long-term side effects [2]. Additionally, 40% of newly diagnosed MB patients develop resistance to therapies and experience recurrence, with TP53-mutant SHH and G3 patients having the poorest survival outcomes and highest relapse rates [3]. Therefore, there is an urgent need for novel treatments that address the heterogeneity of MB and reduce therapy-related morbidities.

Aberrant activation or expression of tyrosine receptor kinases (RTKs) and their ligands is a well-known cause of cancer. Several RTKs, including hepatocyte growth factor receptor (c-MET), platelet-derived growth factor receptor (PDGFR), vascular endothelial growth factor receptor (VEGFR), and ERBB receptors, along with their ligands (HGF, PDGF, VEGF, FGF, and EGF), are implicated in MB pathogenesis [4–8]. These factors initiate signalling cascades, such as MAPK/ERK and PI3K/AKT, that promote the proliferation and survival of MB cells [6, 7]. Recent proteomic studies have demonstrated the aberrant enrichment of multiple RTKs and their downstream intermediaries in MB subgroups [9]. Specifically, Forget and colleagues observed an overall specific activation of ERBB4 and its ligand NRG2 in G4 MB, while Archer and colleagues demonstrated that PRKDC activity is essential for G3 survival after DNA damage [10]. These studies reinforced the idea that aberrant enrichment in the expression of RTK receptors and their ligands is a crucial step in the neoplastic transformation and progression of MB cells, and their inhibition may represent a potential subgroup or subtype-specific therapeutic strategy.

Among the RTKs, c-MET and its ligand HGF play a crucial role in cerebellar development and enhance various malignancy parameters in MB, including mitogenicity, motility, and morphogenesis [11]. HGF and c-MET are expressed in both foetal and adult cerebellum. Activation of the HGF/c-MET signalling pathway stimulates the proliferation of cerebellar granule cell precursors (GCPs) and protects them from apoptosis [12]. Reduced activity of this pathway in mice results in cerebellar abnormalities and reduced GCP proliferation [11]. Conversely, aberrant activation of the c-MET pathway promotes MB cell proliferation, anchorage-independent growth, and cell cycle progression via CDK2/p27 [7]. HGF treatment also protects MB cells from chemotherapy-induced apoptosis by decreasing levels of cleaved PARP and caspase-3 [7]. The oncogenic role of the HGF/c-MET pathway in MB is not typically due to single gene mutations but rather to epigenetic alterations or copy number variations. For example, no activating mutations have been found in the *c-MET* gene, but *HGF* and *c-MET* gene loci on chromosome 7 often show copy number gains in primary MB specimens [13]. Additionally, the *SPINT2* gene, an inhibitor of the HGF/c-MET pathway, is frequently silenced by promoter methylation or deletion in primary MB samples. Re-expression of SPINT2 in MB cell lines with active HGF/c-MET signalling impairs cell proliferation and migration and enhances survival in xenograft models [14]. The therapeutic potential of targeting the HGF/c-MET pathway is supported by in vivo studies showing that *HGF* gene transfer increases MB formation in cerebellar neuronal progenitors in cooperation with SHH signalling [7]. Systemic administration of a monoclonal antibody against HGF has prolonged the survival of xenograft mice, indicating the viability of HGF/c-MET-targeted approaches for treating HGF/c-MET-expressing MB tumours. Small molecule inhibitors against c-MET kinase, which cross the blood-brain barrier more efficiently than antibodies, have also shown promise. Pre-clinical studies of inhibitors like crizotinib [15, 16], SGX523 [17], foretinib [18], and PHA665752 [19] have demonstrated efficacy in reducing the proliferation and motility of MB cells, though their effects vary across different cell lines.

In this study, we evaluated c-MET expression and activity in SHH MB cell lines with varying TP53 statuses and investigated the effects of multiple c-MET inhibitors. Our findings indicate that c-MET inhibitors utilise different mechanisms to halt the cell cycle and reduce cell proliferation in SHH MB cells. Notably, tivantinib exhibited the highest anti-proliferative activity and induced apoptosis in a subset of SHH-MB cells, suggesting it as a promising therapeutic candidate for SHH MB patients.

## Results

### c-MET kinase is preferentially expressed in SHH MB

**Fig. 1:**
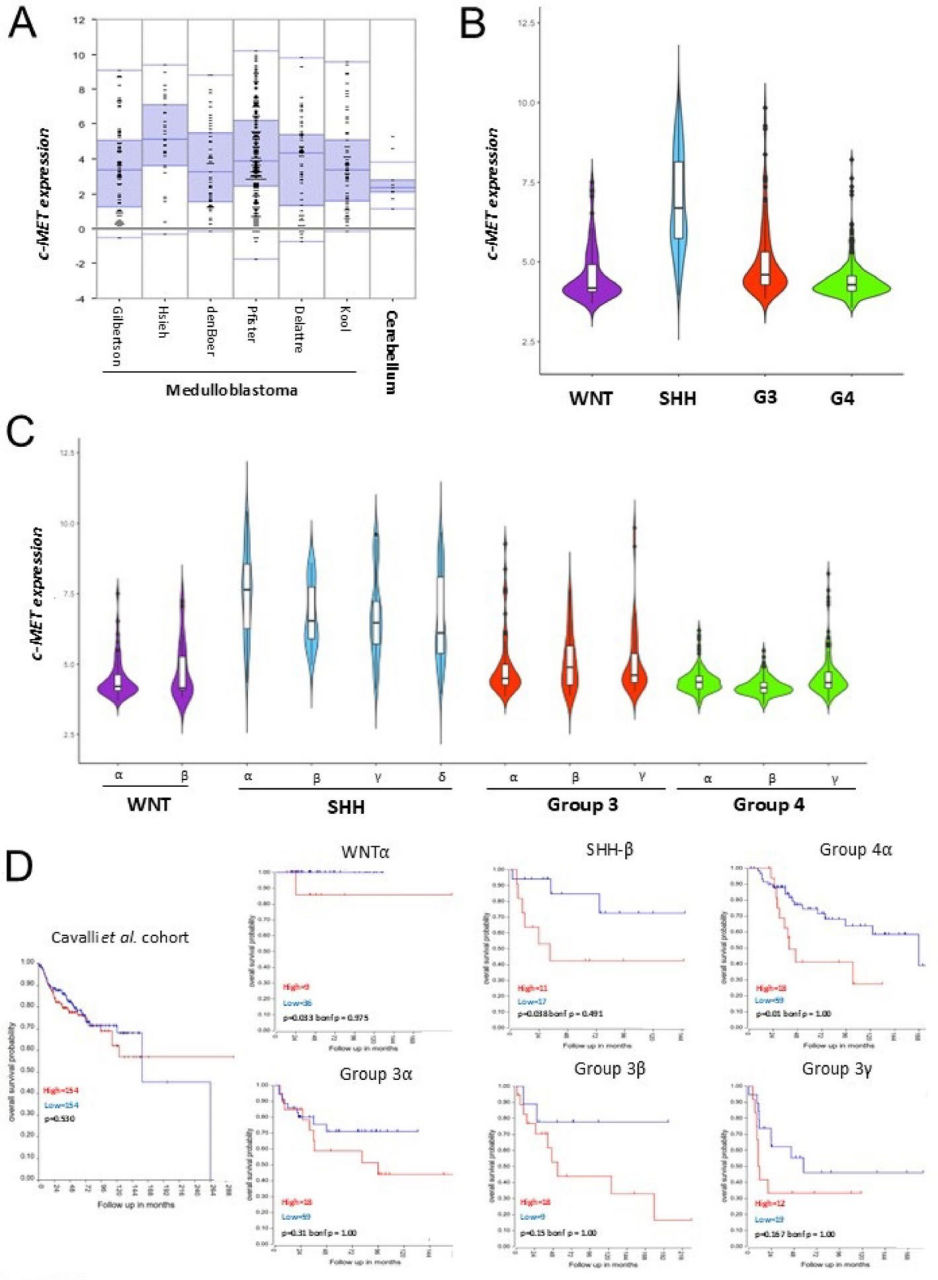
In silico analysis of c-MET expression in MB patients. **A** c-MET Log2 normalised gene expression across six different datasets of MB samples compared with healthy cerebella. Tumour samples comprehend a mix of MB specimens belonging to the different subgroups. One way ANOVA *p*-value = 8.56e-15 (no. of samples: Gilbertson *n* = 76; Hsieh *n* = 31; den Boer *n* = 51; Pfister *n* = 223; Delattre *n* = 57; Kool *n* = 62; Roth *n* = 9). **B** c-MET Log2 normalised gene expression in Cavalli dataset (MB samples = 763) across MB subgroups. One way ANOVA *p*-value: 1.01e-115 (no. of samples: WNT = 70; SHH = 223; Group 3 = 144; Group 4 = 326). **C** c-MET Log2 normalised gene expression across subtypes of MB. One way ANOVA *p*-value: 3.75e-115 (no. of samples: WNTα = 21; WNTβ = 49; SHHα = 65; SHHβ = 35; SHHγ = 76; SHHδ = 47; Group 3α = 67; Group 3β = 37; Group 3γ = 40; Group 4α = 98; Group 4β = 109; Group 4γ = 119). **D** Kaplan-Meier survival curves based on c-MET expression (high and low) were generated on R2 software across the Cavalli cohort of MB patients. High- and low- c-MET expressing cohorts were separated based on Kaplan Scan tool in R2 visualisation platform. Raw *p*-values and Bonferroni corrections for multiple comparison are represented

The c-MET/HGF axis has been proposed as a potential target for treating SHH MB [18, 20]. To confirm its activity in SHH MB cells, we assessed the expression of c-MET kinase and the other 56 RTK gene family members in a discovery cohort of 500 MB tumour samples and 9 normal adult cerebella using the publicly available R2 databases (https://r2.amc.nl). Only 29 RTK genes were significantly modulated in MB tumours compared to normal cerebella (Fig. S1, Supplemental Table S1). Of these, only 7 genes (*c-MET*,* RYK*,* EPHA3*,* EPHB2*,* PTK7*,* ROR1* and *2*) including *c-MET*, were significantly overexpressed in MB tumours, with only c-MET being druggable with multiple, highly selective, and potent inhibitors (Fig. 1A and Fig. S1). We extended this finding by analysing c-MET kinase expression in MB subgroups and subtypes in an independent validation cohort of 763 primary tumour specimens with clinical data [21]. c-MET expression was highest in SHH MB (Fig. 1B). Among the SHH subtypes, SHH-α, affecting non-infant children (> 3 years old) with germline and somatic TP53 mutations, MYCN or GLI2 amplification, and ELP1 alterations, showed robust c-MET expression (Fig. 1C) [19, 21]. Other SHH subtypes (Shh-β, Shh-γ, Shh-δ) also expressed higher c-MET levels compared to the remaining MB samples (Fig. 1C).

Since c-MET lacks activating mutations in MB [18], the presence of its ligand HGF is required for activating c-MET signalling and has been implicated in promoting MB cell dissemination and invasion [20]. We therefore evaluated HGF expression across MB samples, subgroups, and subtypes. HGF expression was reduced in MB specimens compared with normal cerebella (Fig. S2A), consistent with its predominant production by highly differentiated granule and Purkinje cells that are lost in MB tumours [12]. HGF was preferentially expressed in SHH MB tumours and subtypes (Fig. S2B, C). However, no positive correlation between c-MET and GF expression was observed in SHH MB tumours or in WNT subgroup specimens. Instead, only a weak positive association between c-MET and HGF expression was detenced in a limited subset of MB specimens (< 30%), more frequently among G3 and G4 patients (Fig. S2D).

Additionally, we examined c-MET expression in a publicly available single cell RNA sequencing dataset comprising 6,775 cells from 25 MB patients (Fig. S3A) [22]. Only 12.5% of the cells expressed c-MET (Fig. S3B, C). High levels of c-MET expression were observed in cells from all three SHH-subgroup patients, including one sample identified as a metastatic lesion. In contrast, only one G3 patient exhibited relatively high levels of c-MET, and this was confined to a restricted population of cells. No expression of c-MET was detected in fibroblasts (Fig. S3B). Interestingly, HGF expression was either absent or relatively low across the dataset, and only 0.15% of cells co-expressed both c-MET and HGF (Fig. S3C). This further supports the notion that HGF-driven c-MET signalling in MB primarily originates from the normal cerebellar parenchyma.

Finally, we examined the correlation between overall survival and c-MET expression in MB patients. Survival differences were not significant across the full cohort, subgroups or subtypes (Fig. 1D). No significant correlation or positive trend to survival was observed between HGF expression and patient survival (Fig. S12A, B). Overall, these results confirm that c-MET kinase is overexpressed in MB compared to the normal cerebellum, primarily in SHH MB specimens, providing a rationale for targeting c-MET kinase in SHH-MB.

### c-MET kinase is active in SHH MB and promotes MB proliferation

**Fig. 2:**
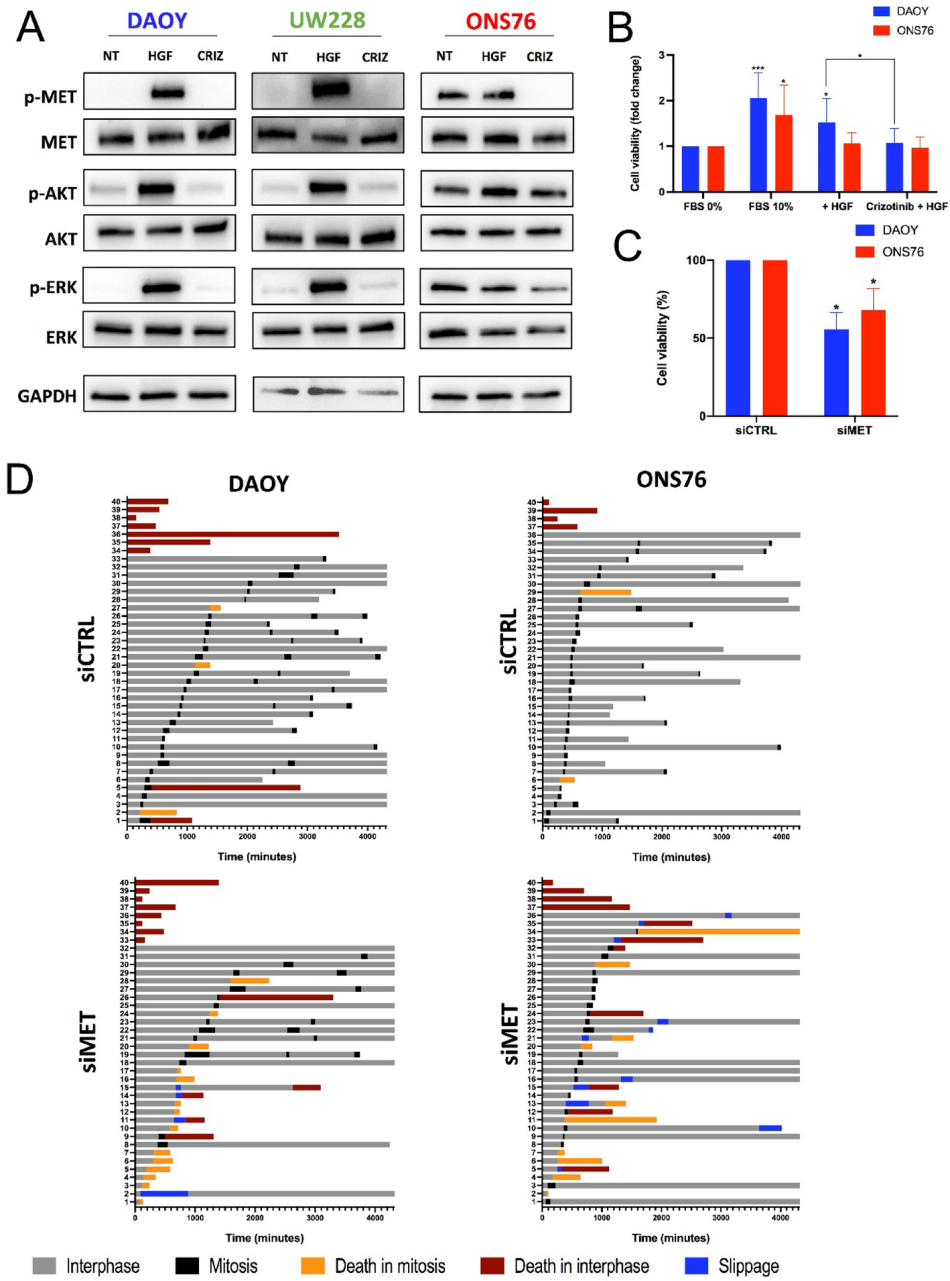
c-MET pathway in SHH medulloblastoma cell lines. **A** Western blot images upon stimulation with HGF (10ng/ml) in SHH medulloblastoma cell lines; cells were starved in 0% FBS media overnight and pre-treated for 2 h with crizotinib (1 µM) prior to stimulation with HGF (10 ng/ml). 40 µg of total lysates were loaded and GAPDH was used as loading control. **B** DAOY and ONS76 were starved with 0% FBS medium overnight. HGF (10 ng/ml) and crizotinib (1 µM) were added for 24 h before MTT assay was performed to assess cell viability. Results are representative of three experiments and are shown as Mean ± SD. Statistical significance was calculated with student’s t-test (*p* ≤ 0.05 = *, *p* ≤ 0.01 = **, *p* ≤ 0.001=***, ns = non-significant). **C** DAOY and ONS76 cells were transiently transfected with 100 nM of siRNA scramble (siCTRL) and siRNA directed against c-MET (siMET). After 72 h, cell viability was assessed by MTT assay. Results are representative of two independent experiments shown as Mean ± SD of eight technical replicates. Significance between siMET and siCTRL was calculated with student’s t-test (*p* ≤ 0.05 = *, *p* ≤ 0.01 = **, *p* ≤ 0.001=***). **D** Cell fate profiles of DAOY and ONS76 transfected with siCTRL and siMET (100nM) for 72 h. Each horizontal bar indicates duration of different phases for a single cell tracked every 15 min for 72 h by time-lapse microscopy. The figure legend representing the different cell fates applies to both cell fate bar graphs. The results, repeated twice, were obtained from eight different technical replicates and 40 representative cells are shown

HGF-dependent c-MET activation leads to receptor dimerization and auto-phosphorylation, modulating downstream pathways like ERK1/2 and PI3K/AKT that affect tumour growth and progression [23]. To assess c-MET pathway integrity in SHH-driven MB cells, we examined the biochemical response of c-MET kinase to HGF in three SHH-MB cell lines. We initially measured c-MET auto-phosphorylation following exogenous HGF stimulation. Western blot analysis showed c-MET auto-phosphorylation was similarly induced by brief HGF exposure (30 min) in DAOY and UW228 cell lines (Fig. 2A). Pre-incubation with crizotinib, a known c-MET inhibitor [24], blocked c-MET tyrosine phosphorylation in both cell lines. Conversely, ONS76 cells showed lower HGF responsiveness: a slight reduction in c-MET auto-phosphorylation after serum starvation suggested ligand-independent or autocrine c-MET phosphorylation [25], followed by limited increased phosphorylation, also blocked by crizotinib. Similar results were observed for downstream signalling (pERK and pAKT) activation after HGF stimulation. Notably, ONS76 cells had high baseline pERK and pAKT levels after serum starvation, with minimal HGF-induced stimulation (Fig. 2A).

We then explored c-MET kinase’s role in SHH MB cell proliferation. First, we assessed MB cell line proliferation upon HGF stimulation. DAOY and ONS76 cells were serum-starved before HGF stimulation (Fig. 2B); starvation halted DAOY cell growth, but 10 ng/mL HGF significantly increased DAOY cell numbers. Co-treating DAOY cells with crizotinib stopped HGF-induced proliferation. HGF did not significantly increase ONS76 cell proliferation, which continued growing under serum starvation due to high basal pAKT and pERK levels (Fig. 2B). Next, we used siRNA-mediated knockdown of c-MET kinase in DAOY and ONS76 cells to examine the effect on proliferation. siRNA targeting c-MET reduced c-MET protein levels (Fig. S4A), significantly decreasing proliferation 72 h post-transfection (Fig. 2C), showing that direct c-MET suppression affects MB cell growth. We further characterised the effects of c-MET knockdown on DAOY and ONS76 cell proliferation by time-lapse microscopy, tracking 40 siRNA-transfected cells over 72 h through at least one mitotic event. Each tracked cell is represented as a single column, with time spent in different cellular states displayed (Fig. 2D). Most control cells divided multiple times, while siRNA targeting c-MET significantly delayed mitosis in both cell lines (Fig. 2D; Fig. S4B). c-MET reduction led to increased death in mitosis and mitotic slippage. DAOY cells showed a 25% increase in mitotic death, compared to 12.5% in ONS76 cells. ONS76 cells had 27.5% mitotic slippage versus 10% in DAOY cells. Of the 14 cells with mitotic slippage: six died in interphase after the first mitosis; two entered interphase but died in the next mitosis; six returned to interphase without further division. Interphase death rates were similar for control and c-MET knockdown cells, likely due to transfection toxicity.

These data confirm SHH MB cells can activate c-MET kinase upon HGF stimulation, relying partially on c-MET signalling for proliferation [26]. Despite similar proliferation reduction after c-MET knockdown, SHH cell lines showed different responses to c-MET signalling alterations, indicating heterogeneous behaviour among SHH MB cell lines to c-MET activity.

### Tivantinib is the most effective c-MET inhibitor against SHH MB cell lines

**Fig. 3:**
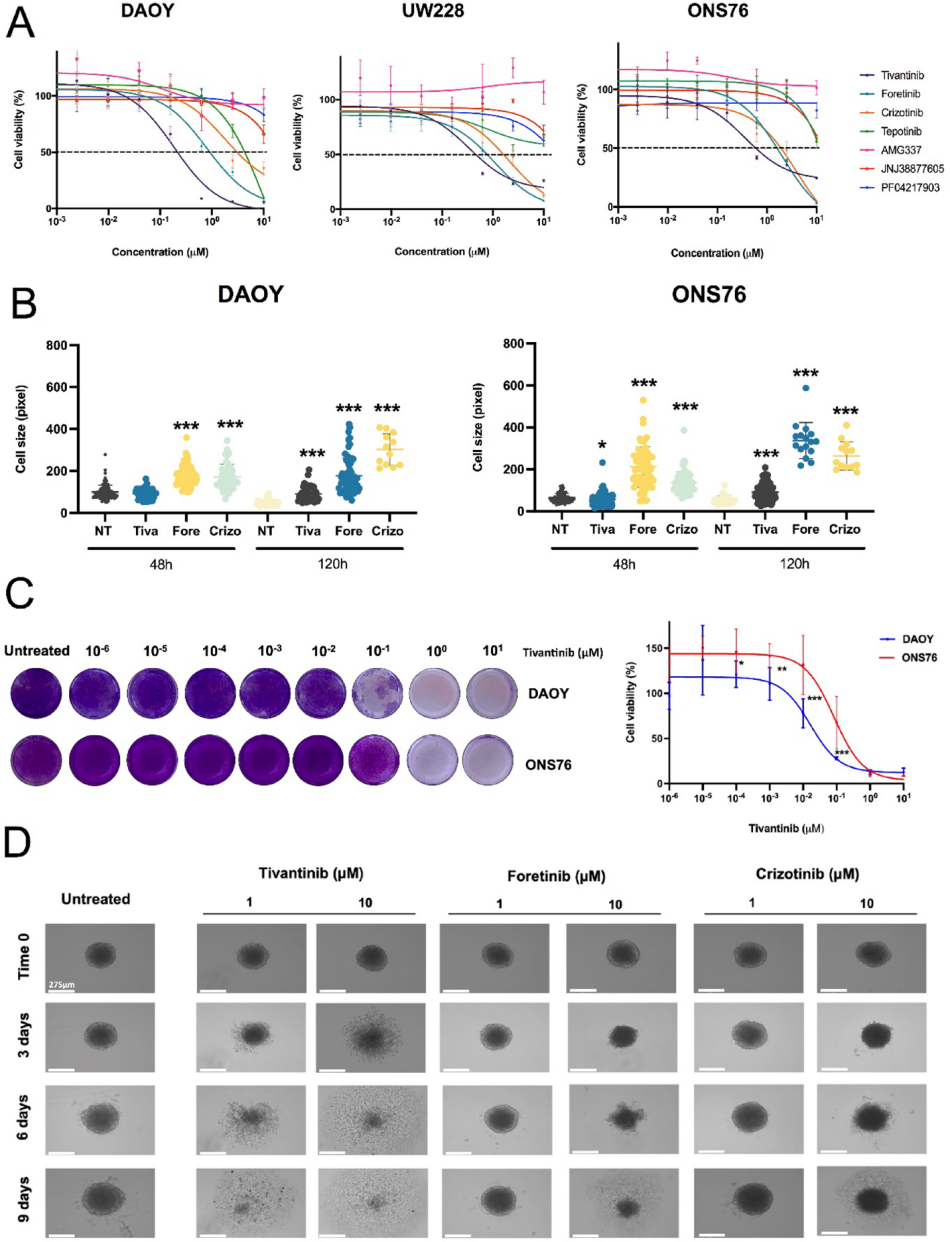
c-MET inhibitors effects on in vitro cultured medulloblastoma cells. **A** Viability curves of three in vitro SHH MB models assessed upon 72 h treatment with increasing concentrations of c-MET kinase inhibitors (fold dilution 1:4 starting at 10 µM). Results are relative to untreated cells and representative of three independent experiments. **B** DAOY and ONS76 were treated with tivantinib (0.5 µM), foretinib (0.5 µM) and crizotinib (2.5 µM) and cell sizes were measured after 48 h and 120 h using ImageJ software. **C** Representative crystal violet pictures of long-term treatment with increasing concentration of tivantinib; media with drug was replaced every 3 days for a total of 9 days. Results are representative of three experiments and relative to untreated cells. **D** Representative pictures of DAOY spheroids treated with tivantinib, foretinib and crizotinib for 9 days with drug’s treatment every 3 days. Pictures were taken with Evos FL Auto 2 microscope (Invitrogen) at 10X magnification (scale bar = 275 μm)

We next evaluated the effectiveness of small molecule c-MET inhibitors on the proliferation of SHH MB cells. SHH MB cells were treated with serial dilutions of seven c-MET inhibitors, each with different potencies and ATP-competitive profiles, and MTT assays were performed 72 h after dosing. Tivantinib, a highly selective, non-ATP competitive, and orally bioavailable inhibitor, was the most effective in reducing cell viability across all three SHH MB cell lines (Fig. 3A; Fig. S5A). Foretinib and crizotinib also demonstrated anti-proliferative activity but were less potent than tivantinib. Bright field microscopy revealed different morphological responses: cells treated with tivantinib accumulated cellular debris and death fragments (Fig. S5B), while cells treated with crizotinib and foretinib showed a drastic increase in cell size with minimal presence of cellular debris (Fig. 3B; Fig. S5B). We further assessed tivantinib’s efficacy by measuring the cell doubling time of DAOY and ONS76 cells. Tivantinib induced a significant, dose- and time-dependent anti-proliferative effect after 24 h. DAOY cell proliferation was completely halted after 72 h, while ONS76 cells showed some residual viability (Fig. S5C). DAOY and ONS76 cells were also subjected to repetitive tivantinib treatments over 9 days, with drug replacement every three days. Crystal violet staining showed increased efficacy, with complete eradication of DAOY cells. However, ONS76 cells formed persistent colonies after 9 days of treatment with tivantinib at 1 and 10µM concentrations (Fig. 3C).

To confirm tivantinib’s efficacy, we used a 3D cell culture model of DAOY cells to assess drug sensitivity compared to foretinib and crizotinib. Spheroids were treated with two concentrations of each inhibitor. Tivantinib reduced spheroid volume after 3 days and completely disintegrated the structures after 9 days (Fig. 3D; Fig. S5D). Crizotinib and foretinib required ten times higher concentrations to impair spheroid growth and were less effective than tivantinib (Fig. 3D; Fig. S5D).

Overall, these results suggest tivantinib is a superior option for inhibiting SHH MB growth compared to foretinib and crizotinib, which have been considered potential clinical options for SHH MB patients [6, 18, 27].

### Tivantinib induces mitotic death in SHH MB cells

**Fig. 4:**
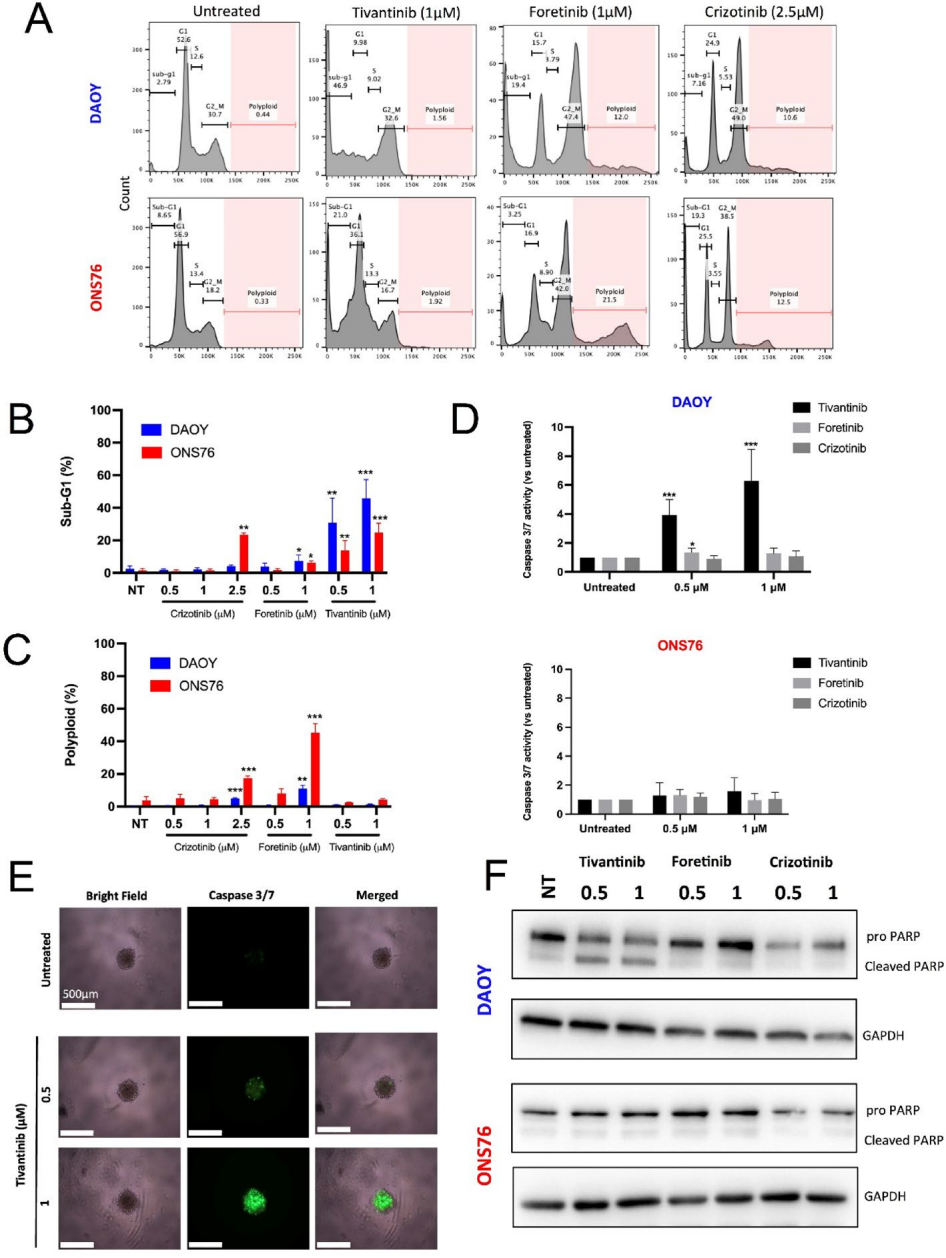
Tivantinib induces apoptotic death in SHH MB cells. **A** Representative histogram plots of DAOY and ONS76 cell cycle treated with tivantinib, foretinib and crizotinib. Cell cycle phases were determined based on DNA content by propidium iodide staining with flowcytometry. **B**,** C** Sub-G1 populations (B) and polyploid (C) were separately calculated, and results are presented as Mean ± SD of three experiments. Statistical comparison was calculated with student’s t-test (*p* ≤ 0.05 = *, *p* ≤ 0.01 = **, *p* ≤ 0.001=***). **D** Caspase 3/7 activation in DAOY and ONS76 cells treated for 24 h with tivantinib, foretinib and crizotinib at different concentrations. Statistical significance was calculated by Student’s t-test (*p* ≤ 0.05 = *, *p* ≤ 0.01 = **, *p* ≤ 0.001=***) between untreated and treated samples. Results are representative of three experiments for DAOY and two experiments for ONS76. **E** Caspase 3/7 activity in DAOY spheroids treated with tivantinib for 24 h. Representative images of three independent experiments with apoptotic cells shown in green were taken with Evos FL Auto 2 microscope at 4X magnification (scale bar = 500 μm). **F** Analysis of full length and cleaved PARP by Western blot in DAOY and ONS76 cells treated with tivantinib, foretinib and crizotinib for 24 h. 40 µg of total lysates were loaded and GAPDH was used as loading control

We investigated whether tivantinib’s anti-proliferative activity in SHH MB cells was associated with apoptosis. Tivantinib induced a dose-dependent accumulation of cells in Sub-G1 for both DAOY and ONS76 cell lines, with DAOY cells showing the highest sensitivity; around 45% of DAOY cells were in sub-G1 after 24 h of treatment (Fig. 4A, B; Fig. S6A). Conversely, foretinib and crizotinib caused significant G2/M accumulation with minor effects on Sub-G1 (Fig. 4A, B; Fig. S6A). Both DAOY and ONS76 cells treated with foretinib and crizotinib exhibited polyploid cell formation (Fig. 4A, C). DAPI staining confirmed increased nuclear size and multinucleation in foretinib and crizotinib treated cells (Fig. S6B, C). We next measured caspase 3/7 activity to explore apoptosis induction. ONS76 cells showed no significant caspase 3/7 activity, whereas DAOY cells exhibited a dose-dependent increase in caspase 3/7 activity only upon tivantinib treatment, also observed in a 3D culture model (Fig. 4D, E). This was supported by the accumulation of cleaved PARP protein in treated DAOY cells (Fig. 4F). Foretinib and crizotinib did not trigger caspase activation or PARP cleavage in either DAOY or ONS76 cells, where significant death was seen only at concentrations 5–10 times higher than the IC50.

**Fig. 5:**
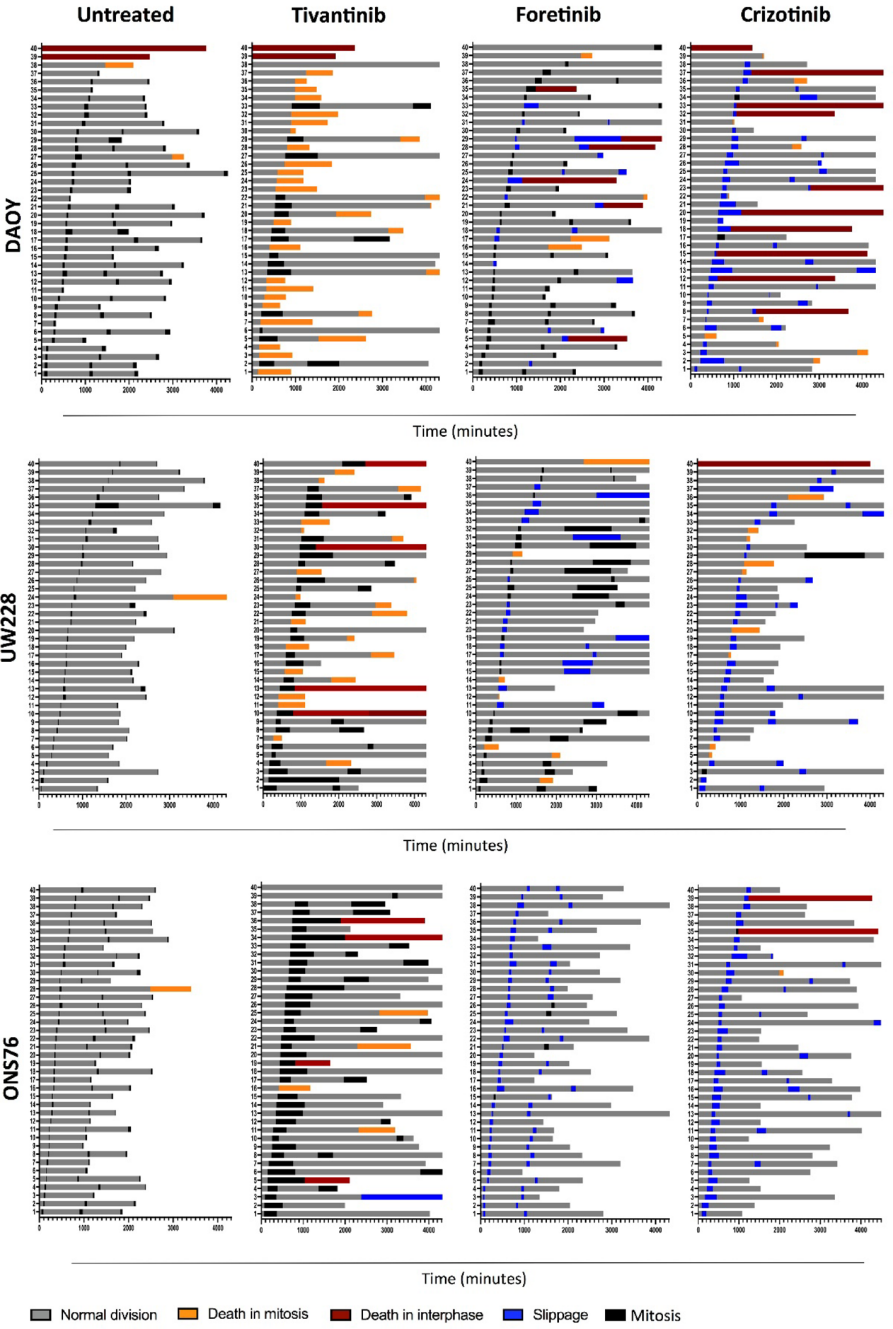
SHH MB cells show heterogeneous responses to different c-MET inhibitors. Cell fate profiles of DAOY, UW228 and ONS76 cells treated with tivantinib, foretinib and crizotinib. Each horizontal bar indicates duration of different phases for a single cell tracked every 15 min for 72 h by time-lapse microscopy. The figure legend represents the different cell fates we observed during imaging analysis. The results, repeated twice, were obtained from 3 different technical replicates and 40 representative cells are shown

To further understand differential response to c-MET inhibitors of SHH MB cells, we tracked again cell fate decisions of DAOY, UW228, and ONS76 cells after treatment with tivantinib, foretinib, and crizotinib. We observed cell fate profiles by tracking 40 unsynchronized cells for 72 h post-treatment. Approximately 95% of untreated cells completed at least one full mitotic division with minimal death in mitosis or interphase (Fig. 5). SHH MB cells showed heterogeneity in response to c-MET inhibitors. Most DAOY cells treated with tivantinib exhibited extended mitosis, with 75% dying in mitosis (Fig. 5, Fig. S7A), whereas all ONS76 cells exited mitosis after prolonged arrest, with only 10% dying in mitosis. Heterogeneity in response to foretinib was also observed. DAOY cells displayed 52% normal fates with multiple mitotic rounds; 40% exhibited slippage, with 22% re-entering the cell cycle and only 18% dying in the following interphase or mitosis (Fig. 5). Foretinib-treated ONS76 cells showed 92% of cells exiting mitosis without division, re-entering the cell cycle but exiting mitosis again without further division in the presence of the drug (Fig. 5). We also evaluated DAOY and ONS76 cell responses to crizotinib. Most DAOY cells treated with crizotinib underwent one or two rounds of mitotic slippage; 25% of slippage events led to cell death in the next interphase or mitosis (Fig. 5). Crizotinib caused mitotic slippage in over 92% of ONS76 cells, with only 5% dying after slippage. UW228 cells treated with tivantinib exhibited extended mitosis followed by mitotic death (Fig. 5, Fig. S7A). When treated with foretinib, 40% of UW228 cells displayed normal fates, 15% showed mitotic death, and 45% underwent slippage. UW228 cells showed an intermediate phenotype between DAOY and ONS76 when treated with crizotinib: 22.5% died in mitosis, while the rest underwent slippage (Fig. 5).

We next assessed the modulation of CCNB1, a key protein in mitotic progression, and the apoptotic inhibitor MCL1 [28]. Tivantinib did not alter CCNB1 levels in DAOY or ONS76 but strongly repressed MCL1 exclusively in DAOY cells (Fig. S7B). DAOY cells showed low CCNB1 levels after foretinib and crizotinib treatment, driving mitotic slippage in the absence of apoptosis. ONS76 cells exhibited increased CCNB1 levels after treatment with foretinib and crizotinib, enabling constant cell cycle re-entry and slippage.

To corroborate the heterogeneous pharmacological response of SHH MB cells to c-MET inhibitors, we generated H2B-GFP positive DAOY and ONS76 cells and tracked their fates using fluorescent imaging upon treatment with tivantinib, foretinib, and crizotinib. We confirmed all cellular fates, including mitotic death in tivantinib-treated DAOY cells, extended mitosis in tivantinib-treated ONS76 cells, and slippage in both cell lines upon foretinib or crizotinib treatment (Fig. S8, S9).

Overall, tivantinib exerts an anti-proliferative effect by inducing a strong apoptotic response after mitotic arrest in 2D and 3D cell culture models of DAOY and UW228 cells. Tivantinib extends mitosis in ONS76 cells without inducing apoptosis at tested concentrations, contrasting with foretinib and crizotinib, which only induce mitotic slippage with limited or absent apoptotic effects.

### Tivantinib synergises with vincristine in affecting SHH medulloblastoma cell proliferation

**Fig. 6:**
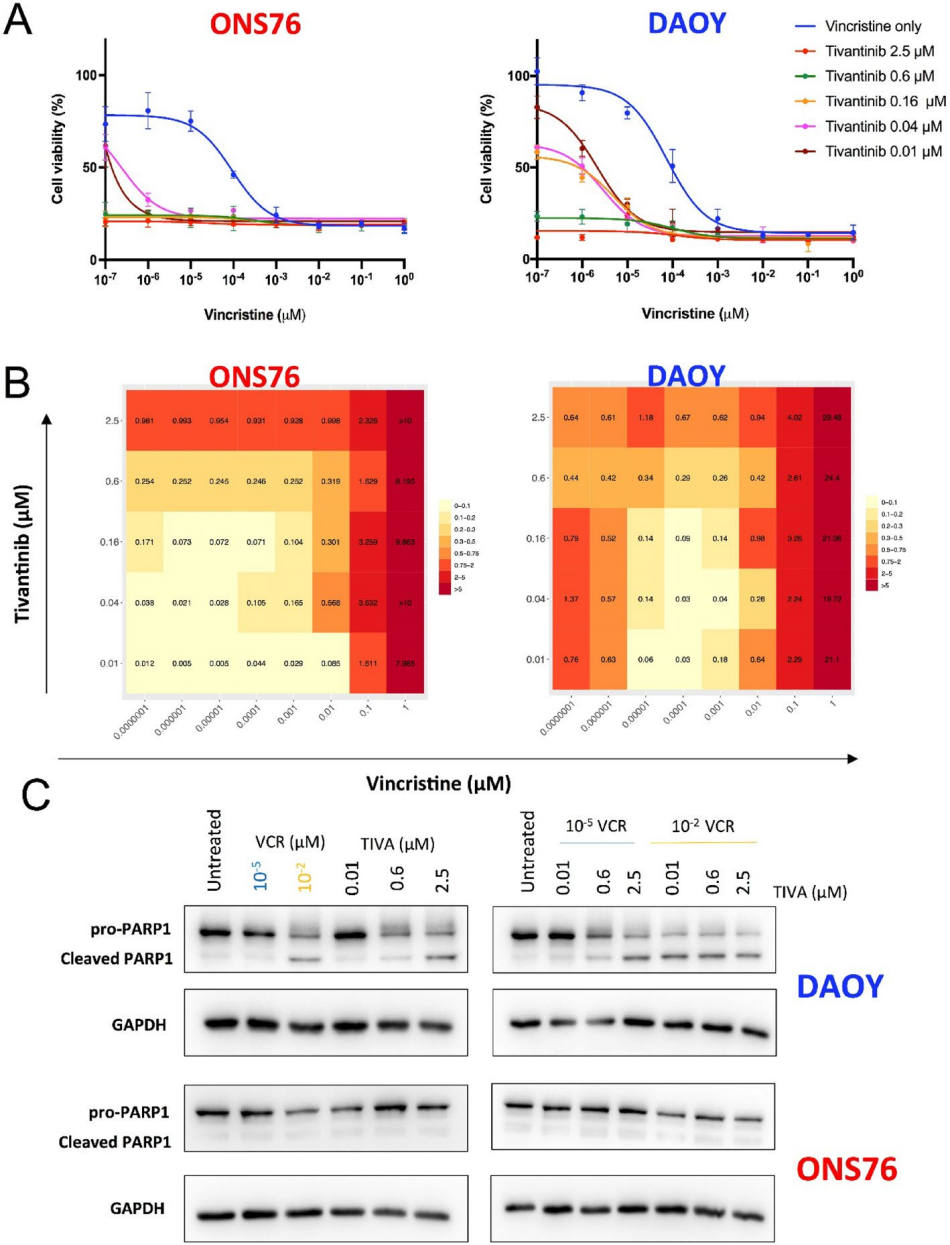
Synergistic effects of tivantinib and vincristine on MB cells. **A** Dose-response curve of DAOY and ONS76 cells treated for 72 h with a combination of vincristine and tivantinib at the indicated concentrations. Results are shown as representative of three experiments. **B** Combination index values were calculated for each combination with Compusyin software (C = 1 additive effect; CI < 1 synergism; C > 1 antagonism). CI values are shown as heatmaps generated with R software. **C** Western blot images performed on DAOY and ONS76 of full length and cleaved PARP following 24 h treatment with combination of vincristine and tivantinib. 40 µg of each sample were loaded and GAPDH was used as loading control

Primary chemotherapy for MB patients includes vincristine, an established antimitotic agent that disrupts microtubule polymerization, leading to mitotic arrest and cell death [29]. Previous findings demonstrated that tivantinib induces mitotic death in TP53 mutant DAOY and UW228 cells, while causing prolonged mitosis in ONS76 cells without triggering apoptosis (Fig. 5). Considering that tivantinib’s antitumor effects involve microtubule inhibition via a mechanism distinct from classical antimitotic drugs [29, 30], we explored its potential to enhance vincristine’s cytotoxicity in both DAOY and ONS76 cell lines. Combination treatments of vincristine and tivantinib were administered to DAOY and ONS76 cells using linear dilutions of vincristine along with five fixed concentrations of tivantinib, covering the IC50 of the drug for both cell lines. Cells were treated with these combinations for 72 h and then subjected to MTT proliferation assays to compare viability percentages with single drug treatments (Fig. 6A). The combined vincristine/tivantinib treatments significantly suppressed the growth of both SHH-MB cell lines compared to single treatments. IC50 analysis for the combinatorial approach in DAOY cells revealed superior efficacy when tivantinib was added to vincristine at concentrations below 0.6 µM. Similarly, ONS76 cells benefited from vincristine/tivantinib combination treatments at tivantinib concentrations of 0.01 and 0.04 µM (Fig. 6A).

Combination indices (CI) were computed using the Chou-Talalay matrix (CI = 1 indicating additive effect, CI < 1 indicating synergism, and CI > 1 indicating antagonism) [31], to evaluate the synergistic interaction of the two drugs. CI analysis confirmed that tivantinib addition sensitized both DAOY and ONS76 cells to vincristine treatments, with the highest synergy observed at low concentrations of both vincristine and tivantinib (Fig. 6B). We also examined the apoptotic response of the combination by measuring PARP1 cleavage levels. Consistent with previous findings, DAOY cells exhibited robust PARP1 cleavage induction after tivantinib-vincristine co-treatment compared to single drug treatments (Fig. 6C). However, the drug combination did not induce PARP1 cleavage in ONS76 cells, indicating the absence of an apoptotic response in this cell line.

Overall, these results suggest a cytotoxic synergy between vincristine and tivantinib specifically in DAOY cells.

### Targeting BCL-2 family induces death in foretinib-treated cells

Following mitotic slippage, a cancer cell can arrest in the next interphase, enter senescence, or die. However, if the cell resumes the cell cycle, it can fuel chromosomal instability, leading to tumour progression and therapy resistance [28, 32, 33]. Given ONS76 cells limited activation of the apoptotic program (low caspase 3/7, low cleaved PARP1, high MCL1) and tendency to undergo multiple rounds of mitotic slippage when treated with c-MET inhibitors (high CCNB1), we hypothesized that the balance between survival and programmed death in this cell line leans towards pro-survival fates, supporting slippage recurrence [34]. To tilt this balance towards cell death, we aimed to enhance the apoptotic trigger by combining foretinib with BCL2-related inhibitors, known to restore apoptosis in cancer cells.

**Fig. 7:**
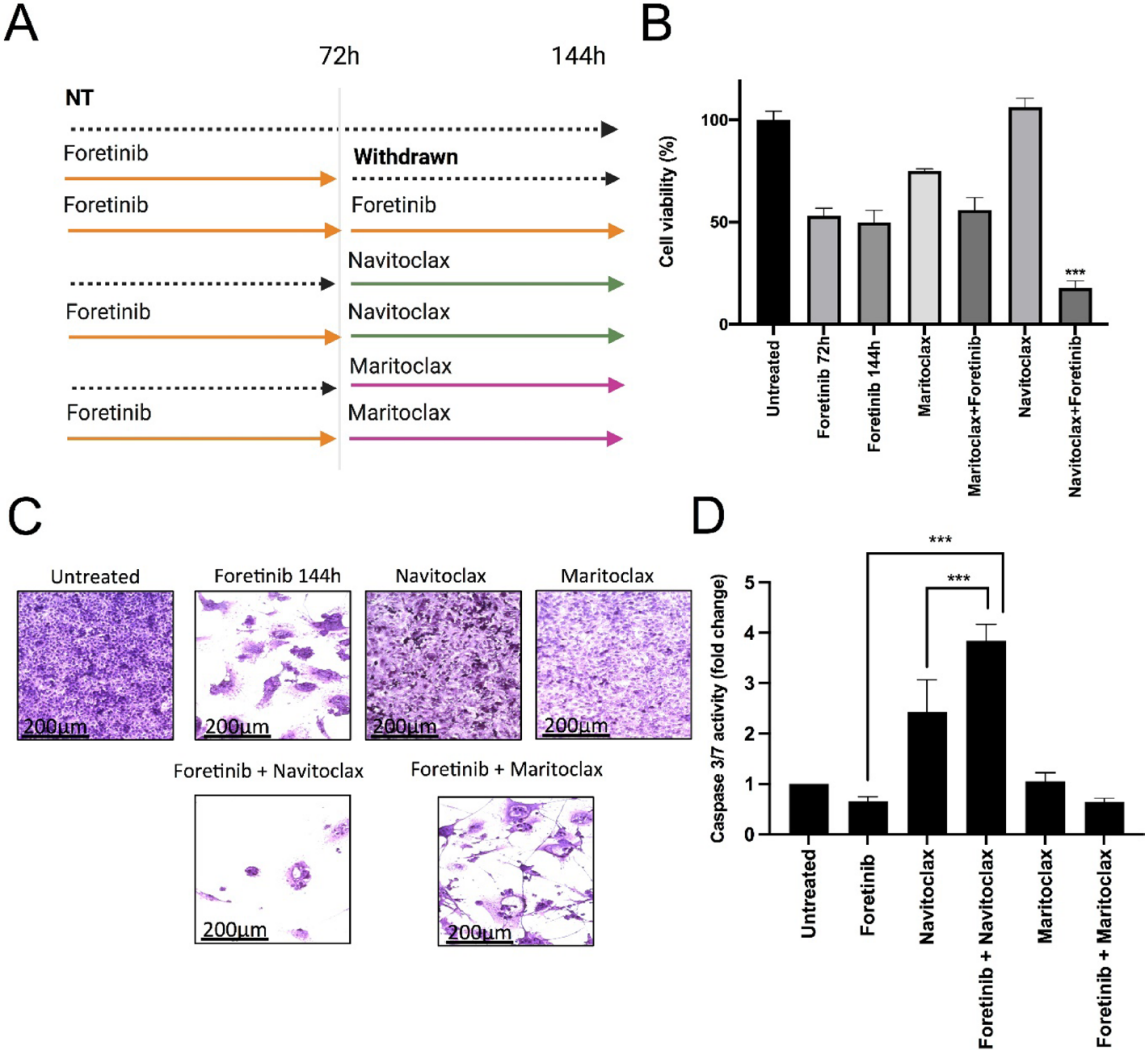
BH3-mimetics induce cell death in mitotic slipped cells. **A** Schematic representation of treatment regimen with foretinib and BH3 inhibitors navitoclax and maritoclax on ONS76 cells. **B** Viability of ONS76 cells pre-treated with foretinib (1 µM) for 72 h before adding navitoclax and maritoclax (2 µM). MTT assay was performed after 72 h. Results are shown as Mean ± SD of two independent experiments. Statistical significance was calculated by Student’s t-test between cells foretinib treatment (72 h and 144 h) and foretinib+navitoclax treatment. (*p* ≤ 0.05 = *, *p* ≤ 0.01 = **, *p* ≤ 0.001=***). **C** Representative crystal violet staining picture showing ONS76 cells treated with sequential regimen of foretinib and BH3 mimetics. **D** Caspase 3/7 activation was assessed in ONS76 cells pre-treated with foretinib (72 h) before adding BH3 mimetics (navitoclax/maritoclax) for 6 h. Results are shown as Mean ± SD and are representative of three experiments. Statistical significance was calculated by Student’s t-test (*p* ≤ 0.05 = *, *p* ≤ 0.01 = **, *p* ≤ 0.001=***)

Initially, we tested the efficacy of maritoclax (MCL-1 inhibitor) and navitoclax (broad BCL-2 family inhibitor) in combination with foretinib. ONS76 cells were treated with linear dilutions of foretinib combined with navitoclax or maritoclax at four different concentrations spanning the drugs’ IC50 [35]. Combinations of foretinib with navitoclax or maritoclax showed synergy, albeit at concentrations too high to be clinically relevant, with synergistic effects observed at 10 µM (Fig. S10A, B). We then investigated whether navitoclax and maritoclax could induce cell death in ONS76 cells after foretinib-induced mitotic slippage. Cells were treated for 72 h with foretinib followed by maritoclax and navitoclax (2 µM) to assess if ‘slipped’ MB cells were more sensitive to inhibition of anti-apoptotic proteins (Fig. 7A). Results indicated that navitoclax was most effective in impairing viability of ONS76 cells pre-treated with foretinib compared to either navitoclax or foretinib alone (Fig. 7B). Conversely, while maritoclax monotherapy affected cell viability, no significant enhancement was observed when used sequentially with foretinib (Fig. 7B). Crystal violet staining revealed the efficacy of navitoclax in sequential treatment with foretinib-pre-treated cells compared to foretinib or navitoclax alone (Fig. 7C, Fig. S10C).

Finally, we assessed navitoclax’s ability to induce cell death by measuring caspase 3/7 activity. ONS76 cells were treated with foretinib (1 µM) for 48 h, followed by navitoclax (2 µM) for 6 h, with untreated and foretinib-treated cells as controls. Activated caspase 3/7 levels increased after navitoclax treatment compared to untreated and foretinib-treated cells (Fig. 7D). These findings suggest that the cytostatic response to foretinib in ONS76 cells can be shifted towards a cytotoxic response by general inhibition of BCL2-family proteins.

## Discussion

This study delineates the significance of the HGF/c-MET pathway in Sonic Hedgehog (SHH) medulloblastoma cells, underscoring the role of c-MET activity in fostering cell proliferation. Two distinct SHH MB cell lines, DAOY and ONS76, were subjected to genetic ablation of c-MET, revealing a dependency on c-MET expression for their proliferative capabilities. Notably, DAOY cells activated the apoptotic program following c-MET knockdown, while ONS76 cells exhibited mitotic slippage with limited cell death. The pharmacological inhibition of c-MET, particularly with tivantinib, a non-competitive ATP inhibitor, demonstrated superior anti-proliferative and cytotoxic effects compared to other c-MET inhibitors like crizotinib or foretinib, previously shown to be effective in killing MB cells [6]. Cell fate analysis and fluorescent chromatin tagging showed that tivantinib preferentially induced mitotic death in TP53-mutant DAOY cell line, whilst ONS76 cells carrying a TP53-wildtype gene experienced extended mitosis with minimal apoptosis [36]. This was a finding further supported by the results on UW228 cells that harbour as well a TP53 mutation. Neither foretinib nor crizotinib stimulated significant cell death across SHH MB cell lines but rather led to mitotic slippage, resulting in increased nuclear size or multinucleated structures. Sequential treatment combining tivantinib with vincristine enhanced cytotoxicity in DAOY cells. Additionally, the BCL2 inhibitor navitoclax activated apoptosis in ONS76 cells, but only when these cells were pre-treated with foretinib, indicating a complex interaction between these agents.

High expression levels of *c-MET* and *HGF* has been previously observed in MB patients and shown to be predictive of an unfavourable prognosis [7]. Here, the analysis of a large-scale omics study involving 763 MB primary MB tumour samples confirmed elevated expression of c-MET kinase and HGF ligand specifically in SHH MB specimens [21]. No significant correlation was found between *c-MET* and *HGF* expression levels in SHH patients. This suggests that c-MET signalling in SHH tumours may not be fully driven by tumour-autologous HGF production. Instead, pathway activation could depend on HGF produced within the cerebellum, where it normally supports granule neuron survival and proliferation [37]. This was further supported by the scRNAseq data showing the absence of co-expression of c-MET and HGF in malignant cells whilst still corroborating the high level of c-MET in SHH patients. Several other growth factors, including epidermal growth factor (EGF), insulin growth factor (IGF-I), and Shh can also stimulate granule cell proliferation in vitro [38–40]. This raises the possibility that c-MET may be indirectly activated through cross-talk with other receptor tyrosine kinases (RTKs) to promote SHH-MB development and progression. In this context, c-MET signalling and homeostasis are known to be modulated by a plethora of c-MET interacting proteins, such as plexins, integrins, semaphorins, or other RTKs [41]. For example, IGF-1 has been shown to induce delayed c-MET activation in prostate cancer cells co-expressing c-MET and IGF-1R, suggesting that IGF-1R-mediated c-MET activation may contribute to tumorigenic processes in various cancer types, an aspect that remains unexplored in medulloblastoma [42]. In contrast to WNT and SHH tumours, G3 and G4 MB specimens showed a weak but statistically detectable correlation between c-MET and HGF expression. However, the strength of this association was modest, and its biological significance remains unclear. Notably, c-MET expression was highest in SHH MB, providing a stronger rationale for therapeutic targeting in this subgroup, which is also the focus of the functional analyses presented here. While the observed c-MET/HGF co-expression in a subset of G3 and G4 tumours may suggest additional context-dependent regulatory mechanisms, any therapeutic implications for these subgroups remain speculative. Whether modulation of c-MET signalling or disruption of the HGF–c-MET interaction influences G3 or G4 tumour cell behaviour will require dedicated functional studies using appropriate subgroup-specific models.

We also investigated the correlation between c-MET expression and the overall survival of MB patients. Unfortunately, high c-MET expression was not predictive of poor outcomes in molecular or histological subgroups, presence of metastasis or gender. Interestingly, only patients belonging to the 10–17 age category (categories: 0–4, 4–10, 10–17, > 18) showed a significant correlation between poor overall survival and c-MET high-expression (Fig. S11). The SHH child category (children above 4 years of age) was previously identified by Clifford group as a carrier of *GLI2* amplification, *MYCN* amplification and *TP53* mutation but, as shown here, man other parameters could be considered to further refine the group [43, 44]. It is important to note that expression of c-MET alone is not predictive of the activation status of c-MET itself. Its phosphorylation status should be inspected for a more robust assessment. In this context, previously published histochemical analysis of p-c-MET confirmed that c-MET activation correlates with increased relapse risk and shorter progression-free survival in paediatric SHH MB patients [43, 45, 46]. Therefore, more in depth exploration at clinical level should be considered for implementing c-MET based therapy for MB, an area still unexplored for paediatric cancer.

Tivantinib demonstrated potent anti-proliferative activity linked to cytotoxic effects on SHH MB cells in both 2D and 3D cultures. It extended mitotic time and induced mitotic death in DAOY and UW228 cells but not in ONS76 cells, which showed prolonged mitosis without apoptotic death. The observed synergy between tivantinib and vincristine in killing DAOY cells suggests that tivantinib’s mechanisms of action may extend beyond c-MET inhibition. Indeed, previous research indicates tivantinib can disrupt microtubule polymerization via direct tubulin interaction, similarly to vincristine, inducing a G2/M cell cycle arrest [47, 48]. This dual action on c-MET and microtubules may account for the observed enhanced anti-proliferative effects. Beyond c-MET, tivantinib targets other pathways, including eNOD-like receptor (NLR) family pyrin domain containing 3 (NLRP3) and VEGFR signaling [49, 50]. An unbiased proteomic approach identified tivantinib as a potent inhibitor of GSK3α and GSK3β, more so than c-MET [47]. This inhibition of GSK3 kinases, particularly GSK3α, contributes to PARP cleavage and G2/M cell cycle arrest, further supporting tivantinib’s role in anti-cancer activity through multiple pathways. The interaction between c-MET/HGF signaling and the Wnt/β-catenin axis, coupled with tivantinib’s ability to inhibit microtubule polymerization, suggests that tivantinib can maximize therapeutic efficacy against c-MET dependent MB. Tivantinib’s broader kinase inhibition profile, compared to other c-MET inhibitors such as tepotinib, MK-2461, and golvantinib, makes it a potent option for treating SHH MB. Although crizotinib shows higher c-MET biochemical activity suppression, tivantinib exhibits superior anti-proliferative activity across multiple cell lines, including lung and thyroid cancer cells [47, 51].

In early studies, cancer patients associated with high levels of c-MET expression or c-MET mutations benefited from tivantinib [52, 53]. Despite these early positive results, tivantinib encountered significant challenges in larger and later clinical trials. Two major phase 3 studies in hepatocellular carcinoma (HCC) patients failed to meet their primary endpoints of prolonging overall survival or progression-free survival, leading to the discontinuation of the tivantinib program for HCC and other indications [54, 55]. Up to today, tivantinib has not received approval for any indication in any country as a stand-alone therapy. However, several clinical trials II showed that tivantinib in combination with other TKI could significantly improve survival and overcome acquired resistance [52]. In addition, a phase-I clinical trial showed that tivantinib is a well-tolerated drug that can be administered in children with paediatric tumours, including MB, in tablet or powder form when taken with food [56]. Interestingly, one out of four MB patients demonstrated a positive response to the treatment, suggesting a potential, but as yet unvalidated, therapeutic benefit in a subset of patients. Therefore, potential use of tivantinib in combination with other TKI inhibitors or chemotherapeutic drugs could be explored as new avenues for the treatment of SHH-MB.

Finally, Foretinib has previously been shown to suppress MB cell proliferation and improve survival in mouse xenograft and transgenic models of SHH MB [18]. However, in our experiments, foretinib and crizotinib exhibited lower anti-proliferative activities and limited impact on mitotic time and cell death in SHH MB cells compared to tivantinib. Treatment with foretinib and crizotinib resulted in mitotic slippage with minimal cell death, leading to the formation of multinucleated and polyploid cells. This polyploidy could contribute to genetic instability, aneuploidy, and cancer progression, underscoring the need to shift from mitotic slippage to apoptotic death for effective MB therapy [57–59]. Blocking anti-apoptotic proteins like MCL1 or BCL2 can tip the balance towards apoptosis, providing a potential strategy to eliminate genomically unstable and polyploid MB cells. Sequential treatment of foretinib-treated ONS76 cells with the pan-BCL2 inhibitor navitoclax resulted in increased apoptosis and eradication of these cells, highlighting a promising approach to overcome treatment resistance and prevent disease progression [28, 34, 60].

In conclusion, tivantinib demonstrates a multifaceted mechanism of action, combining c-MET inhibition with effects on microtubule dynamics and potential GSK3 kinase inhibition. This versatility supports its repurposing as a therapeutic agent for SHH MB, particularly in combination with vincristine to reduce toxicity and enhance efficacy. Alternatively, foretinib in combination with BCL2 inhibitors like navitoclax could serve as an effective strategy to prevent mitotic slippage and polyploidy, ultimately improving clinical outcomes for SHH MB patients. In both cases, while pre-clinical findings are promising, further validation will require extensive experimentation, including in vitro studies and, more importantly, in vivo evaluation of efficacy and PK/PD using patient-derived xenografts (PDX) and other relevant SHH MB models.

## Materials and methods

*Cell culture.* DAOY cell line was purchased from the American Tissue Culture Collection (ATCC) [35, 61]. ONS76 and UW228 were kindly provided by Prof. Caroline Springer [35, 62]. Cells were grown in RPMI-1640 (Biosera) supplemented with 10% fetal bovine serum (FBS) and 1% penicillin/streptomycin (Gibco). Cells were maintained at 37 C in a humidified atmosphere and 5% CO_2_ and passaged by trypsinisation when at 80% confluency. DAOY 3D spheroids were generated by seeding cells in Nunclon Sphera U-bottom 96-well microplates and incubated for five days in humidified atmosphere at 37 C and 5% CO_2_ before drug treatment.

*Tumour gene expression data.* For the RTK data analysis, medulloblastoma tumours and normal cerebellum gene expression datasets were retrieved from R2 Genomics Analysis and Visualization Platform (http://r2.amc.nl). Referenced accession numbers for the studies: Gilbertson, GSE37418; Hsieh, GSE67851; denBoer, GSE74195; Pfister, Pubmed link: 28,726,821; Delattre, R2 internal identified: ps_avgpres_mbdelatrepublic57_u133p2; Kool, GSE10327; normal cerebellum, Roth, GSE3526. The correlation analysis between c-MET and HGF expression in subgroups and subtypes and survival analysis were performed using an independent cohort of 612 samples (Cavalli, GSE85217) [21]. Kaplan Scan is used to define high- and low- cMET expressing patients where the best survival cut-off is identified by statistical testing (see R2: Genomics Analysis and Visualization Platform). scRNAseq dataset (SCAR_Atlas_0679) were retrieved from Single Cell and Spatially resolved Cancer Resources (SCAR) and analysed by using the SCAR_Atlas portal [22, 63].

*Cell viability assay.* DAOY (1500 cells/well), ONS76 (1000 cells/well) and UW228 (2000 cells/well) were seeded in 96-well plates and 24 h later treated with MET inhibitors. Post-treatment images were taken after 72 h at 10 X magnification with an EVOS FL Auto 2 microscope. Thiazolyl Blue Tetrazolium bromide (MTT) assays were performed after 72 h of treatment and absorbance recorded at 540/630 nm with a Varioskan^tm^ LUX multimode microplate reader. For long-term proliferation assays, DAOY and ONS76 cells were seeded at low density (150 cells/well) in 96-well plate and cells were treated with drug every 3 days for a total of 9 days; crystal violet staining was performed by exposing cells to 0.5% (w/v) crystal violet solution for 15 min and pictures were taken before dissolution and quantification.

*Cell cycle analysis by Flow Cytometry.* DAOY and ONS76 cells were treated in six well plates for 24 h with MET inhibitors. Cells were detached with trypsin, washed twice with PBS, fixed in cold ethanol 70% and stored at -20 C for at least 24 h before staining. Cells were stained with propidium iodide (PI) solution (50 µg/ml) supplemented with 5 µg RNaseA (APExBIO). 10.000 events for each sample were recorded by using a BD FACSVerse flow cytometer. FlowJo software was used to analyse and plot cell cycle profiles.

*Caspase 3/7 activation.* Apoptotic cells in 2D culture were detected by using the Caspase-Glo 3/7 Assay kit (Promega) following the manufacture’s protocol and luminescence recorded by using a Varioskan^tm^ LUX multimode microplate reader. Detection of apoptotic cells in 3D DAOY-derived spheroids was performed by using the CellEvent Caspase-3/7 Green Detection Reagent (ThermoFisher). Spheroids were treated with drugs for 24 h and fluorescent reagents added for 30 min before fluorescence images were taken by using an Evos FL Auto 2 microscope.

*siRNA Transient transfection.* ON-TARGET SMARTpool siRNA against c-MET (Cat.ID: L-003156-00-0005) and siGENOME non-Targeting siRNA Pool (Cat.ID: L-001810-01-05) were purchased from Dharmacon and resuspended in DNase/RNase-free water at 20 µM stock concentration. Cells were transfected by using Lipofectamin 2000 (Invitrogen) with siRNA molecules (100nM) dissolved in Reduced-Serum Medium Opti-MEM (Gibco) and cells maintained in antibiotic-free media. Cell culture media was replaced 24 h after transfection. Data for MTT and cell fates were collected from eight technical replicates and the experiment was repeated twice.

*Generation of stable H2B-eGFP cell lines.* H2B-GFP plasmid harbouring the neomycin resistance gene (Addgene, #11680) was purified by using Qiagen Plasmid Midi Kit and transfected in DAOY and ONS76 cells (2 µg) plated in 6 well plates by using Lipofectamin 2000 in a ratio 1:3 of DNA to lipofectamin. Transfected DAOY and ONS76 cells were kept in media enriched with neomycin (Roche) at 1 mg/mL concentration for 2 weeks and the presence of GFP-positive cells was confirmed by microscopy before performing further analyses.

*Cell fate analysis by time-lapse microscopy.* Cell fates analysis was performed as previously described in Buzzetti et al. [35]. ONS76, DAOY and UW228 cells were seeded in a 96 well plate and 24 h later treated with c-MET inhibitors. Plates were immediately subjected to imaging by using a Cytation 3 system (Biotek): cells were maintained at 37 °C and 5% CO_2_ and pictures were taken every 15 min for 72 h at 100X magnification. For siRNA-mediated knockdown experiments, transfected cells were subjected to imaging 24 h after transfection and when cell culture media was replaced. 40 cells for each condition were manually analysed by looking at changes in cell morphology to define different cell cycle phases over time. Knockdown experiments were performed twice with similar results. At time 0, only cells in interphase (with a flat morphology) were taken further for the analysis. Interphase duration, mitotic time and time of cell death were recorded and plotted using GraphPad Prism 8. When presented in the text, the percentage of cells in a specific fate was calculated as follows: (number of cells in a specific fate)/(Total number of tracked cells). All cell fate experiments were repeated twice with similar results, and 40 representative cells from multiple technical replicates are depicted in the figures.

*Western blot analysis.* Cell pellets were lysed in triple lysis buffer (50 mM Tris-HCl (pH 7.5), 150 mM NaCl, 0.1% SDS, 1% NP-40 and 0.5% sodium deoxycholate) with protease and phosphatases inhibitors (Santa Cruz Biotechnology, USA). Protein lysates (40 µg) were subjected to SDS-PAGE electrophoresis and then transferred to PVDF membranes. Blots were blocked in 5% milk, PBS-Tween 0.1% solution and incubated with primary antibodies overnight at 4 C followed by one hour incubation with HRP-conjugated secondary antibodies raised in rabbit or mouse (anti-Rabbit IgG from Cell Signalling Technologies, 1:5000; anti-Mouse IgG from GE Healthcare, 1:3000). Band visualisation was completed by using West Femto Chemilumiscent Substrate (ThermoFisher, UK) and images were recorded by using a G: BOX SynGene system. GAPDH was used as loading control. The following primary antibodies (Cell signalling, UK) were used for the immunoblot detection: MET (#4562, 1:1000), phospho-MET (#3077, 1:1000), p44/p42 MAPK (#4695, 1:1000), phospho-p44/42 MAPK (#9101, 1:1000), AKT (#9272, 1:1000), phospho-AKT (#4060. 1:1000), Cyclin B1 (#4138, 1:1000), PARP (#9542, 1:1000) MCL-1 (#4572, 1:1000). Anti-GAPDH antibody was purchased from MerckMillipore (#AB2302, 1:10,000).

*Statistical analysis.* Data analyses and graphs for all cellular experiments were completed with GraphPad Prism Software 9. Differences between two experimental groups were determined by using a two-tailed student’s t-test (*p* ≤ 0.05 = *, *p* ≤ 0.01 = **, *p* ≤ 0.001=***) and data represented as media ± SD. Difference in gene expression between tumour and normal cerebellum were determined by corrected *p*-value (Benjamini-Hochberg, *p*-value < 0.05). Log-rank test was used in the survival analysis with respect to c-MET and HGF expression between subgroups and subtypes of MB patients: high and low expression cohort were generated based on median gene expression.

## Supplementary Information

Below is the link to the electronic supplementary material.

Supplementary Material 1 ( Supplementary Table 1. Dysregulated TKI genes with an increased expression in MB tumours compared to normal adult cerebellum.).

Supplementary Material 2 (Supplementary Fig. 1. Gene expression analysis of tyrosine kinase receptors derived from six different publicly available datasets (Gilbertson n = 76; Hsieh n = 31; denBoer n = 51; Pfister n = 223; Delattre n = 57; Kool n = 62) compared with expression in healthy cerebellum (Roth n = 9). Heatmap representation of gene expression is based on log2 normalised gene expression signal of all analysed RTK. Only seven genes (RYK, ROR1, ROR2, EPHB2, PTK7, EPHA3, MET) out of 29 significantly modulated RTK genes (FLT3, AATYK, FGFR1, TYRO3, KIT, FGFR3, NTRK2, EPHA4, FGFR2, ROS1, RYK, EPHB3, ROR1, EPHB2, STYK1, PTK7, RON, EPHA5, ERBB3, CSF1R, EPHA3, ALK, MET, EPHA7, INSRR, RET, ROR2, PDGFRB, VEGFR3) showed an increased expression in tumour compared to normal (corrected p-value < = 0.05). Heatmap was generated by plotting gene expression values on R software. Red arrows indicate genes significantly overexpressed in MB compared to normal adult cerebella. Tumour samples comprehend a mix of MB specimens belonging to the different subgroups. Supplementary Fig. 2. (A) HGF Log2 normalised expression across six different datasets of medulloblastoma samples compared with healthy cerebella (One way ANOVA p-value = 5.53x10− 16) (Gilbertson n = 76; Hsieh n = 31; den Boer n = 51; Pfister n = 223; Delattre n = 57; Kool n = 62; Roth n = 9). Tumour samples comprehend a mix of MB specimens belonging to the different subgroups. (B) HGF Log2 normalised gene expression in Cavalli dataset (MB samples = 612) across medulloblastoma subgroups (no of samples: WNT = 70; SHH = 223; Group 3 = 144; Group 4 = 326). One way ANOVA p-value = 3.27e-26. (C) HGF Log 2 normalised gene expression across subtypes of medulloblastoma (no of samples: WNT = 21; WNT = 49; SHH = 65; SHH = 35; SHH =  76; SHH = 47; Group 3 =  67; Group 3 = 37; Group 3 = 40; Group 4 =  98; Group 4 = 109; Group 4 = 119). One way ANOVA p-value = 8.77e-34. (D) Correlation between HGF and c-MET expression (from Cavalli et al. dataset). Supplementary Fig. 3. (A) A publicly available scRNAseq dataset comprising 6,775 cells from a cohort of 25 MB patients was queried to assess the expression of c-MET and HGF. UMAP shows cells according to sample of origin (WNT: SJ516, MUV44, BCH807, SJ99, SJ129; SHH: MUV41, SJ454, SJ577; G3: MUV11, SJ17, SJ917, SJ617, MUV29, BCH1205, MUV34, BCH825; G4: SJ625, MUV39, SJ723, SJ217, MUV27, BCH1031, MUV19, MUV37, SJ970), malignant versus normal (fibroblast) cell distribution and disease state (primary and metastatic lesion). (B) UMAP showing the expression of c-MET across the cell population. (C) Dotplot showing the expression of cMET and HGF according to subgroups. (D) Expression of both cMET (red) and HGF (blue) is represented in the UMAP to assess co-expression of the receptor and ligand. Only 0.15% of the cells shows co-expression of both genes. Supplementary Fig. 4. (A) Western blot images to confirm successful knockdown of c-MET gene via siRNA in DAOY and ONS76. GAPDH is used as a loading control. (B) Densitometric analysis of the MET knockdown in DAOY and ONS76 cells. MET expression is normalized to GAPDH. (C) Time in mitosis for both DAOY and ONS76 cells calculated for each single cell tracked by time-lapse imaging. Time in mitosis is expressed in minutes and it is the time a cell spends to complete a full mitotic division. Supplementary Fig. 5. (A) Summary table of IC50 calculated for each c-MET inhibitors using GraphPad Software. Values are presented as Mean ± SD of three independent experiments. (B) Representative images of DAOY and ONS76 treated with crizotinib, foretinib and tivantinib at 2.5 M for 72 h. Pictures were taken at Evos FL Auto 2 microscope at 10X (scale bar = 275 m). Red arrows indicate enlarged cells; white arrows indicate cell death fragments. (C) Growth rates analysis performed on DAOY and ONS76 cells upon 72 h treatment with tivantinib at different concentrations. Results are relative to time 0 and are shown as representative of three independent experiments. (D) Representative growth rates of DAOY-derived spheroids (relative to images shown in Fig. 3D) after treatment with tivantinib, foretinib and crizotinib. Results are shown as Mean ± SD. Statistical significance was calculated with student’s t-test (p ≤ 0.05 = *, p ≤ 0.01 = **, p ≤ 0.001=***, ns = non-significant). Supplementary Fig. 6. (A) Representative percentages of DAOY and ONS76 cells gated in G1, S and G2/M phases post 24 h treatment with crizotinib, foretinib and tivantinib. (B) Images of DAPI stained DAOY and ONS76 cells upon 72 h treatment with tivantinib (0.5 M) Foretinib (0.5 M) and Crizotinib (2.5 M). Pictures were taken at Evos FL Auto 2 microscope at 20X (scale bar = 100 m). (C) Nuclear sizes of DAOY and ONS76 cells were measured post c-MET inhibitors treatments using ImageJ Software. Statistical significance was calculated with student’s t-test (p ≤ 0.001=***). Supplementary Fig. 7. (A) Time in mitosis for DAOY, UW228 and ONS76 cells were calculated for each single cell tracked by time-lapse imaging during drug treatments. Time in mitosis is expressed in minutes and it is the time a cell spend to complete a full mitotic division. (B) Analysis of CCNB2 and MCL-1 by Western blot in DAOY and ONS76 cells treated with tivantinib, foretinib and crizotinib for 24 h. 40 µg of total lysates were loaded and GAPDH was used as loading control. Supplementary Fig. 8. Representative pictures of different cell fate profiles in H2B-eGFP expressing-DAOY cells upon treatment with tivantinib, foretinib and crizotinib. Pictures were taken by using a Cytation 3 system (Biotek) (scale bar = 100 m) and time frame is represented below each picture. Supplementary Fig. 9. Representative pictures of different cell fate profiles in H2B-eGFP expressing-ONS76 cells upon treatment with tivantinib, foretinib and crizotinib. Pictures were taken by using a Cytation 3 system (Biotek) (scale bar = 100 m) and time frame is represented below each picture. Supplementary Fig. 10. Dose-response curve of ONS76 cells treated for 72 h with a combination of foretinib and either navitoclax or maritoclax at the indicated concentrations. (B) Combination index values were calculated for each combination with Compusyin software (C = 1 additive effect; CI < 1 synergism; C > 1 antagonism). CI values are shown as heatmaps generated with R software. (C) Representative image of crystal violet staining on ONS76 cells treated with 1 M foretinib for 72 h. Supplementary Fig. 11. Overall survival of MB patients stratified based on age. The Cavalli cohort was divided in four age groups (0–4, 4–10, 10–17, > 18) and overall survival assessed based on c-MET expression. High- and low- c-MET expressing cohorts were separated based on Kaplan Scan tool in R2 visualisation platform. Raw p-values and Bonferroni corrections for multiple comparison are represented. Supplementary Fig. 12. Kaplan-Meier survival curves based on HGF expression (high and low) were generated on R2 software across the Cavalli cohort of MB patients: (A) full cohort of patients; (B) MB subgroups. High- and low- HGF expressing cohorts were separated based on Kaplan Scan tool in R2 visualisation platform. Raw p-values are represented.).

## Data Availability

Medulloblastoma and normal cerebellum gene expression datasets were retrieved from R2 Genomics Analysis and Visualization Platform (http://r2.amc.nl). Referenced accession numbers for the studies: Gilbertson, GSE37418; Hsieh, GSE67851; denBoer, GSE74195; Pfister, Pubmed link: 28726821; Delattre, R2 internal identified: ps_avgpres_mbdelatrepublic57_u133p2; Kool, GSE10327; normal cerebellum, Roth, GSE3526. The correlation analysis between c-MET and HGF expression in subgroups and subtypes and survival analysis were performed using an independent cohort of 612 samples (Cavalli, GSE85217) [21]. scRNAseq dataset (SCAR_Atlas_0679) were retrieved from Single Cell and Spatially resolved Cancer Resources (SCAR) and analysed by using the SCAR_Atlas portal [22,63]. 21. Cavalli FMG, Remke M, Rampasek L, Peacock J, Shih DJH, Luu B, et al. Intertumoral Heterogeneity within Medulloblastoma Subgroups. Cancer Cell 2017;31:737-754.e6. 10.1016/j.ccell.2017.05.005.22. Hovestadt V, Smith KS, Bihannic L, Filbin MG, Shaw ML, Baumgartner A, et al. Resolving medulloblastoma cellular architecture by single-cell genomics. Nature 2019;572:74–9. 10.1038/s41586-019-1434-6.63. Deng Y, Chen P, Xiao J, Li M, Shen J, Qin S, et al. SCAR: Single-cell and Spatially-resolved Cancer Resources. Nucleic Acids Res 2024;52:D1407–17. 10.1093/nar/gkad753.

## References

[1] Louis DN, Perry A, Wesseling P, Brat DJ, Cree IA, Figarella-Branger D, et al. The 2021 WHO Classification of Tumors of the Central Nervous System: a summary. Neuro Oncol. 2021;23:1231–51. 10.1093/neuonc/noab106. 34185076 10.1093/neuonc/noab106 PMC8328013

[2] Hovestadt V, Ayrault O, Swartling FJ, Robinson GW, Pfister SM, Northcott PA. Medulloblastomics revisited: biological and clinical insights from thousands of patients. Nat Rev Cancer. 2020;20:42–56. 10.1038/s41568-019-0223-8. 31819232 10.1038/s41568-019-0223-8 PMC9113832

[3] Hill RM, Richardson S, Schwalbe EC, Hicks D, Lindsey JC, Crosier S, et al. Time, pattern, and outcome of medulloblastoma relapse and their association with tumour biology at diagnosis and therapy: a multicentre cohort study. Lancet Child Adolesc Health. 2020;4:865–74. 10.1016/S2352-4642(20)30246-7. 33222802 10.1016/S2352-4642(20)30246-7 PMC7671998

[4] Ajeawung NF, Wang HY, Gould P, Kamnasaran D. Advances in molecular targets for the treatment of medulloblastomas. Clin Invest Med. 2012;35:E246. 10.25011/cim.v35i5.18697. 23043706 10.25011/cim.v35i5.18697

[5] Penco-Campillo M, Comoglio Y, Feliz Morel ÁJ, Hanna R, Durivault J, Leloire M, et al. VEGFC negatively regulates the growth and aggressiveness of medulloblastoma cells. Commun Biol. 2020;3:579. 10.1038/s42003-020-01306-4. 33067561 10.1038/s42003-020-01306-4 PMC7568583

[6] Zomerman WW, Plasschaert SLA, Diks SH, Lourens H-J, Meeuwsen-de Boer T, Hoving EW, et al. Exogenous HGF Bypasses the Effects of ErbB Inhibition on Tumor Cell Viability in Medulloblastoma Cell Lines. PLoS ONE. 2015;10:e0141381. 10.1371/journal.pone.0141381. 26496080 10.1371/journal.pone.0141381 PMC4619778

[7] Li Y, Lal B, Kwon S, Fan X, Saldanha U, Reznik TE, et al. The Scatter Factor/Hepatocyte Growth Factor: c-Met Pathway in Human Embryonal Central Nervous System Tumor Malignancy. Cancer Res. 2005;65:9355–62. 10.1158/0008-5472.CAN-05-1946. 16230398 10.1158/0008-5472.CAN-05-1946

[8] Gilbertson RJ, Langdon JA, Hollander A, Hernan R, Hogg TL, Gajjar A, et al. Mutational analysis of PDGFR–RAS/MAPK pathway activation in childhood medulloblastoma. Eur J Cancer. 2006;42:646–9. 10.1016/j.ejca.2005.11.023. 16434186 10.1016/j.ejca.2005.11.023

[9] Archer TC, Ehrenberger T, Mundt F, Gold MP, Krug K, Mah CK, et al. Proteomics, Post-translational Modifications, and Integrative Analyses Reveal Molecular Heterogeneity within Medulloblastoma Subgroups. Cancer Cell. 2018;34:396–e4108. 10.1016/j.ccell.2018.08.004. 30205044 10.1016/j.ccell.2018.08.004 PMC6372116

[10] Forget A, Martignetti L, Puget S, Calzone L, Brabetz S, Picard D, et al. Aberrant ERBB4-SRC Signaling as a Hallmark of Group 4 Medulloblastoma Revealed by Integrative Phosphoproteomic Profiling. Cancer Cell. 2018;34:379–395.e7. 10.1016/j.ccell.2018.08.002. 10.1016/j.ccell.2018.08.002 30205043

[11] Desole C, Gallo S, Vitacolonna A, Montarolo F, Bertolotto A, Vivien D, et al. HGF and MET: from Brain Development to Neurological Disorders. Front Cell Dev Biol. 2021;9:683609. 10.3389/fcell.2021.683609. 34179015 10.3389/fcell.2021.683609 PMC8220160

[12] Ieraci A, Forni PE, Ponzetto C. Viable hypomorphic signaling mutant of the Met receptor reveals a role for hepatocyte growth factor in postnatal cerebellar development. Proceedings of the National Academy of Sciences. 2002;99:15200–5. 10.1073/pnas.222362099 10.1073/pnas.222362099 PMC137567 12397180

[13] Hui AB-Y, Lo K-W, Yin X-L, Poon W-S, Ng H-K. Detection of Multiple Gene Amplifications in Glioblastoma Multiforme Using Array-Based Comparative Genomic Hybridization. Lab Invest. 2001;81:717–23. 10.1038/labinvest.3780280. 11351043 10.1038/labinvest.3780280

[14] Kongkham PN, Northcott PA, Ra YS, Nakahara Y, Mainprize TG, Croul SE, et al. An Epigenetic Genome-Wide Screen Identifies *SPINT2* as a Novel Tumor Suppressor Gene in Pediatric Medulloblastoma. Cancer Res. 2008;68:9945–53. 10.1158/0008-5472.CAN-08-2169. 19047176 10.1158/0008-5472.CAN-08-2169

[15] Zhang Y, Farenholtz KE, Yang Y, Guessous F, diPierro CG, Calvert VS, et al. Hepatocyte Growth Factor Sensitizes Brain Tumors to c-MET Kinase Inhibition. Clin Cancer Res. 2013;19:1433–44. 10.1158/1078-0432.CCR-12-2832. 23386689 10.1158/1078-0432.CCR-12-2832 PMC3602223

[16] Michieli P, Mazzone M, Basilico C, Cavassa S, Sottile A, Naldini L, et al. Targeting the tumor and its microenvironment by a dual-function decoy Met receptor. Cancer Cell. 2004;6:61–73. 10.1016/j.ccr.2004.05.032. 15261142 10.1016/j.ccr.2004.05.032

[17] Guessous F, Zhang Y, diPierro C, Marcinkiewicz L, Sarkaria J, Schiff D, et al. An Orally Bioavailable c-Met Kinase Inhibitor Potently Inhibits Brain Tumor Malignancy and Growth. Anticancer Agents Med Chem. 2010;10:28–35. 10.2174/1871520611009010028. 20015006 10.2174/1871520611009010028 PMC3278215

[18] Faria CC, Golbourn BJ, Dubuc AM, Remke M, Diaz RJ, Agnihotri S, et al. Foretinib Is Effective Therapy for Metastatic Sonic Hedgehog Medulloblastoma. Cancer Res. 2015;75:134–46. 10.1158/0008-5472.CAN-13-3629. 25391241 10.1158/0008-5472.CAN-13-3629

[19] Garcia-Lopez J, Kumar R, Smith KS, Northcott PA. Deconstructing Sonic Hedgehog Medulloblastoma: molecular Subtypes, Drivers, and Beyond. Trends Genet. 2021;37:235–50. 10.1016/j.tig.2020.11.001. 33272592 10.1016/j.tig.2020.11.001

[20] Binning MJ, Niazi T, Pedone CA, Lal B, Eberhart CG, Kim KJ, et al. Hepatocyte Growth Factor and Sonic Hedgehog Expression in Cerebellar Neural Progenitor Cells Costimulate Medulloblastoma Initiation and Growth. Cancer Res. 2008;68:7838–45. 10.1158/0008-5472.CAN-08-1899. 18829539 10.1158/0008-5472.CAN-08-1899 PMC2638505

[21] Cavalli FMG, Remke M, Rampasek L, Peacock J, Shih DJH, Luu B, et al. Intertumoral Heterogeneity within Medulloblastoma Subgroups. Cancer Cell. 2017;31:737–e7546. 10.1016/j.ccell.2017.05.005. 28609654 10.1016/j.ccell.2017.05.005 PMC6163053

[22] Hovestadt V, Smith KS, Bihannic L, Filbin MG, Shaw ML, Baumgartner A, et al. Resolving medulloblastoma cellular architecture by single-cell genomics. Nature. 2019;572:74–9. 10.1038/s41586-019-1434-6. 31341285 10.1038/s41586-019-1434-6 PMC6754173

[23] Birchmeier C, Birchmeier W, Gherardi E, Vande Woude GF. Met, metastasis, motility and more. Nat Rev Mol Cell Biol. 2003;4:915–25. 10.1038/nrm1261. 14685170 10.1038/nrm1261

[24] Zou HY, Li Q, Lee JH, Arango ME, McDonnell SR, Yamazaki S, et al. An Orally Available Small-Molecule Inhibitor of c-Met, PF-2341066, Exhibits Cytoreductive Antitumor Efficacy through Antiproliferative and Antiangiogenic Mechanisms. Cancer Res. 2007;67:4408–17. 10.1158/0008-5472.CAN-06-4443. 17483355 10.1158/0008-5472.CAN-06-4443

[25] Rong S, Segal S, Anver M, Resau JH, Vande Woude GF. Invasiveness and metastasis of NIH 3T3 cells induced by Met-hepatocyte growth factor/scatter factor autocrine stimulation. Proc Natl Acad Sci. 1994;91:4731–5. 10.1073/pnas.91.11.4731. 10.1073/pnas.91.11.4731 PMC43862 8197126

[26] Gherardi E, Birchmeier W, Birchmeier C, Woude G Vande. Targeting MET in cancer: rationale and progress. Nat Rev Cancer. 2012;12:89–103. 10.1038/nrc3205. 22270953 10.1038/nrc3205

[27] Endersby R, Whitehouse J, Pribnow A, Kuchibhotla M, Hii H, Carline B, et al. Small-molecule screen reveals synergy of cell cycle checkpoint kinase inhibitors with DNA-damaging chemotherapies in medulloblastoma. Sci Transl Med. 2021. 10.1126/scitranslmed.aba7401. 33472956 10.1126/scitranslmed.aba7401 PMC8994821

[28] Sloss O, Topham C, Diez M, Taylor S. Mcl-1 dynamics influence mitotic slippage and death in mitosis. Oncotarget. 2016;7:5176–92. 10.18632/oncotarget.6894. 26769847 10.18632/oncotarget.6894 PMC4868679

[29] Jordan M. Mechanism of Action of Antitumor Drugs that Interact with Microtubules and Tubulin. Curr Med Chemistry-Anti-Cancer Agents. 2012;2:1–17. 10.2174/1568011023354290. 10.2174/1568011023354290 12678749

[30] Wu Z-X, Yang Y, Teng Q-X, Wang J-Q, Lei Z-N, Wang J-Q, et al. Tivantinib, A c-Met Inhibitor in Clinical Trials, Is Susceptible to ABCG2-Mediated Drug Resistance. Cancers (Basel). 2020;12:186. 10.3390/cancers12010186. 31940916 10.3390/cancers12010186 PMC7017082

[31] Chou T-C. Drug Combination Studies and Their Synergy Quantification Using the Chou-Talalay Method. Cancer Res. 2010;70:440–6. 10.1158/0008-5472.CAN-09-1947. 20068163 10.1158/0008-5472.CAN-09-1947

[32] Sinha D, Duijf PHG, Khanna KK. Mitotic slippage: an old tale with a new twist. Cell Cycle. 2019;18:7–15. 10.1080/15384101.2018.1559557. 30601084 10.1080/15384101.2018.1559557 PMC6343733

[33] Brito DA, Rieder CL. Mitotic Checkpoint Slippage in Humans Occurs via Cyclin B Destruction in the Presence of an Active Checkpoint. Curr Biol. 2006;16:1194–200. 10.1016/j.cub.2006.04.043. 16782009 10.1016/j.cub.2006.04.043 PMC2749311

[34] Topham CH, Taylor SS. Mitosis and apoptosis: How is the balance set? Curr Opin Cell Biol. 2013;25:780–5. 10.1016/j.ceb.2013.07.003. 23890995 10.1016/j.ceb.2013.07.003

[35] Buzzetti M, Morlando S, Solomos D, Mehmood A, Cox AWI, Chiesa M, et al. Pre-therapeutic efficacy of the CDK inhibitor dinaciclib in medulloblastoma cells. Sci Rep. 2021;11:5374. 10.1038/s41598-021-84082-3. 33686114 10.1038/s41598-021-84082-3 PMC7940474

[36] Ivanov DP, Coyle B, Walker DA, Grabowska AM. In vitro models of medulloblastoma: choosing the right tool for the job. J Biotechnol. 2016;236:10–25. 10.1016/j.jbiotec.2016.07.028. 27498314 10.1016/j.jbiotec.2016.07.028

[37] Zhang L, Himi T, Morita I, Murota S. Hepatocyte growth factor protects cultured rat cerebellar granule neurons from apoptosis via the phosphatidylinositol-3 kinase/Akt pathway. J Neurosci Res. 2000;59:489–96. 10679787 10.1002/(SICI)1097-4547(20000215)59:4<489::AID-JNR3>3.0.CO;2-9

[38] Wallace VA. Purkinje-cell-derived Sonic hedgehog regulates granule neuron precursor cell proliferation in the developing mouse cerebellum. Curr Biol. 1999;9:445–8. 10.1016/S0960-9822(99)80195-X. 10226030 10.1016/s0960-9822(99)80195-x

[39] Wechsler-Reya RJ, Scott MP. Control of Neuronal Precursor Proliferation in the Cerebellum by Sonic Hedgehog. Neuron. 1999;22:103–14. 10.1016/S0896-6273(00)80682-0. 10027293 10.1016/s0896-6273(00)80682-0

[40] Dahmane N, Altaba AR. i. Sonic hedgehog regulates the growth and patterning of the cerebellum. Development 1999;126:3089–100. 10.1242/dev.126.14.3089. 10.1242/dev.126.14.3089 10375501

[41] Viticchiè G, Muller P. c-Met and Other Cell Surface Molecules: interaction, Activation and Functional Consequences. Biomedicines. 2015;3:46–70. 10.3390/biomedicines3010046. 28536399 10.3390/biomedicines3010046 PMC5344229

[42] Varkaris A, Gaur S, Parikh NU, Song JH, Dayyani F, Jin J, et al. Ligand-independent activation of MET through IGF‐1/IGF‐1R signaling. Int J Cancer. 2013;133:1536–46. 10.1002/ijc.28169. 23526299 10.1002/ijc.28169 PMC3713179

[43] Ellison DW, Kocak M, Dalton J, Megahed H, Lusher ME, Ryan SL, et al. Definition of Disease-Risk Stratification Groups in Childhood Medulloblastoma Using Combined Clinical, Pathologic, and Molecular Variables. J Clin Oncol. 2011;29:1400–7. 10.1200/JCO.2010.30.2810. 20921458 10.1200/JCO.2010.30.2810 PMC3525837

[44] Schwalbe EC, Lindsey JC, Nakjang S, Crosier S, Smith AJ, Hicks D, et al. Novel molecular subgroups for clinical classification and outcome prediction in childhood medulloblastoma: a cohort study. Lancet Oncol. 2017;18:958–71. 10.1016/S1470-2045(17)30243-7. 28545823 10.1016/S1470-2045(17)30243-7 PMC5489698

[45] Zhukova N, Ramaswamy V, Remke M, Pfaff E, Shih DJH, Martin DC, et al. Subgroup-Specific Prognostic Implications of *TP53* Mutation in Medulloblastoma. J Clin Oncol. 2013;31:2927–35. 10.1200/JCO.2012.48.5052. 23835706 10.1200/JCO.2012.48.5052 PMC4878050

[46] Northcott PA, Hielscher T, Dubuc A, Mack S, Shih D, Remke M, et al. Pediatric and adult sonic hedgehog medulloblastomas are clinically and molecularly distinct. Acta Neuropathol. 2011;122:231–40. 10.1007/s00401-011-0846-7. 21681522 10.1007/s00401-011-0846-7 PMC4538327

[47] Remsing Rix LL, Kuenzi BM, Luo Y, Remily-Wood E, Kinose F, Wright G, et al. GSK3 Alpha and Beta Are New Functionally Relevant Targets of Tivantinib in Lung Cancer Cells. ACS Chem Biol. 2014;9:353–8. 10.1021/cb400660a. 24215125 10.1021/cb400660a PMC3944088

[48] Xiang Q, Zhen Z, Deng DY, Wang J, Chen Y, Li J, et al. Tivantinib induces G2/M arrest and apoptosis by disrupting tubulin polymerization in hepatocellular carcinoma. J Experimental Clin Cancer Res. 2015;34:118. 10.1186/s13046-015-0238-2. 10.1186/s13046-015-0238-2 PMC4603939 26458953

[49] Kim BJ, Kim YJ, Sohn S-H, Kim B, Sul HJ, Kim HS, et al. Tivantinib inhibits the VEGF signaling pathway and induces apoptosis in gastric cancer cells with c-MET or VEGFA amplification. Invest New Drugs. 2020;38:1633–40. 10.1007/s10637-020-00940-3. 32361789 10.1007/s10637-020-00940-3

[50] Huang Y, Guo Y, Zhou Y, Huang Q, Ru Y, Luo Y, et al. Tivantinib alleviates inflammatory diseases by directly targeting NLRP3. IScience. 2023;26:106062. 10.1016/j.isci.2023.106062. 36843841 10.1016/j.isci.2023.106062 PMC9950949

[51] Zhou Y, Zhao C, Gery S, Braunstein GD, Okamoto R, Alvarez R, et al. Off-Target Effects of c-MET Inhibitors on Thyroid Cancer Cells. Mol Cancer Ther. 2014;13:134–43. 10.1158/1535-7163.MCT-13-0187. 24170771 10.1158/1535-7163.MCT-13-0187 PMC3947168

[52] Lu F, Zhao K, Ye M, Xing G, Liu B, Li X, et al. Efficacy and safety of second-line therapies for advanced hepatocellular carcinoma: a network meta-analysis of randomized controlled trials. BMC Cancer. 2024;24:1023. 10.1186/s12885-024-12780-y. 39160484 10.1186/s12885-024-12780-y PMC11331808

[53] Zhao S, Wu W, Jiang H, Ma L, Pan C, Jin C, et al. Selective Inhibitor of the c-Met Receptor Tyrosine Kinase in Advanced Hepatocellular Carcinoma: No Beneficial Effect With the Use of Tivantinib? Front Immunol. 2021. 10.3389/fimmu.2021.731527. 34804015 10.3389/fimmu.2021.731527 PMC8600564

[54] Kudo M, Morimoto M, Moriguchi M, Izumi N, Takayama T, Yoshiji H, et al. A randomized, double-blind, placebo‐controlled, phase 3 study of tivantinib in Japanese patients with MET‐high hepatocellular carcinoma. Cancer Sci. 2020;111:3759–69. 10.1111/cas.14582. 32716114 10.1111/cas.14582 PMC7541009

[55] Rimassa L, Assenat E, Peck-Radosavljevic M, Pracht M, Zagonel V, Mathurin P, et al. Tivantinib for second-line treatment of MET-high, advanced hepatocellular carcinoma (METIV-HCC): a final analysis of a phase 3, randomised, placebo-controlled study. Lancet Oncol. 2018;19:682–93. 10.1016/S1470-2045(18)30146-3. 29625879 10.1016/S1470-2045(18)30146-3

[56] Geller JI, Perentesis JP, Liu X, Minard CG, Kudgus RA, Reid JM, et al. A phase 1 study of the c-Met inhibitor, tivantinib (ARQ197) in children with relapsed or refractory solid tumors: a Children’s Oncology Group study phase 1 and pilot consortium trial (ADVL1111). Pediatr Blood Cancer. 2017. 10.1002/pbc.26565. 28449393 10.1002/pbc.26565 PMC5657151

[57] Liu J. The dualistic origin of human tumors. Semin Cancer Biol. 2018;53:1–16. 10.1016/j.semcancer.2018.07.004. 30040989 10.1016/j.semcancer.2018.07.004 PMC6553492

[58] Duesberg P, McCormack A. Immortality of cancers. Cell Cycle. 2013;12:783–802. 10.4161/cc.23720. 23388461 10.4161/cc.23720 PMC3610726

[59] Salmina K, Huna A, Kalejs M, Pjanova D, Scherthan H, Cragg MS, et al. The Cancer Aneuploidy Paradox: in the Light of Evolution. Genes (Basel). 2019;10:83. 10.3390/genes10020083. 30691027 10.3390/genes10020083 PMC6409809

[60] Bennett A, Sloss O, Topham C, Nelson L, Tighe A, Taylor SS. Inhibition of Bcl-xL sensitizes cells to mitotic blockers, but not mitotic drivers. Open Biol. 2016. 10.1098/rsob.160134. 27512141 10.1098/rsob.160134 PMC5008013

[61] Jacobsen PF, Jenkin DJ, Papadimitriou JM. Establishment of a Human Medulloblastoma Cell Line and Its Heterotransplantation into Nude Mice. J Neuropathol Exp Neurol. 1985;44:472–85. 10.1097/00005072-198509000-00003. 2993532 10.1097/00005072-198509000-00003

[62] Yamada M, Shimizu K, Tamura K, Okamoto Y, Matsui Y, Moriuchi S, et al. Establishment and biological characterization of human medulloblastoma cell lines. No Shinkei. 1989;41:695–702. 2818910

[63] Deng Y, Chen P, Xiao J, Li M, Shen J, Qin S, et al. SCAR: Single-cell and Spatially-resolved Cancer Resources. Nucleic Acids Res. 2024;52:D1407–17. 10.1093/nar/gkad753. 37739405 10.1093/nar/gkad753 PMC10767865

