# Supplementary material for: Distinct cMET inhibitors uncover pharmacological heterogeneity in SHH medulloblastoma cell lines
Source: Discov Oncol. 2026 Feb 23;17:506. doi: 10.1007/s12672-026-04717-7 (PMC13035958; doi:10.1007/s12672-026-04717-7)
Supplement: Supplementary file 1 — Supplementary Material 1 ( Supplementary Table 1. Dysregulated TKI genes with an increased expression in MB tumours compared to normal adult cerebellum.). [file 12672_2026_4717_MOESM1_ESM.pdf]

Morlando et al. Supplemental Table 1. Dysregulated TKI genes with an increased expression in MB tumours compared to normal adult cerebellum.

Genes	Normal Cerebellum (n = 9) average expression	Tumours (n = 500) Average expression	Adjusted p-value
FLT3	6.7	3.54	6E-05
AATYK	9	6.7	1E-04
FGFR1	9.8	7.7	1E-04
TYRO3	8.7	6.2	1E-04
KIT	9.1	6.5	2E-04
FGFR3	8.7	5	6E-04
NTRK2	8.8	7	7E-04
EPHA4	8	6.3	8E-04
FGFR2	8.7	5.7	2E-03
ROS1	4.7	3.1	5E-03
RYK	7.5	8.8	7E-03
EPHB3	6.9	5.4	1E-02
ROR1	4.25	6.3	1E-02
EPHB2	3.6	6.7	3E-02
STYK1/DKFZp761P1010	5.1	3.6	3E-02
PTK7	4.5	6.5	3E-02
RON	5.7	4.2	4E-02
EPHA6	4.3	2.8	4E-02
ERBB3	6.4	4.5	4E-02
CSF1R	7.1	6.2	4E-02
EPHA3	2.7	6.1	4E-02
ALK	6	4.6	4E-02
MET	2.4	3.9	5E-02
EPHA7	7.3	6.5	5E-02
INSRR	4.2	2.2	5E-02
RET	6	4.6	5E-02
ROR2	2.4	3.5	5E-02
PDGFRB	6.88	6	5E-02
VEGFR3	5.3	4.1	5E-02
