# Supplementary figures and images for: Distinct cMET inhibitors uncover pharmacological heterogeneity in SHH medulloblastoma cell lines
Source: Discov Oncol. 2026 Feb 23;17:506. doi: 10.1007/s12672-026-04717-7 (PMC13035958; doi:10.1007/s12672-026-04717-7)

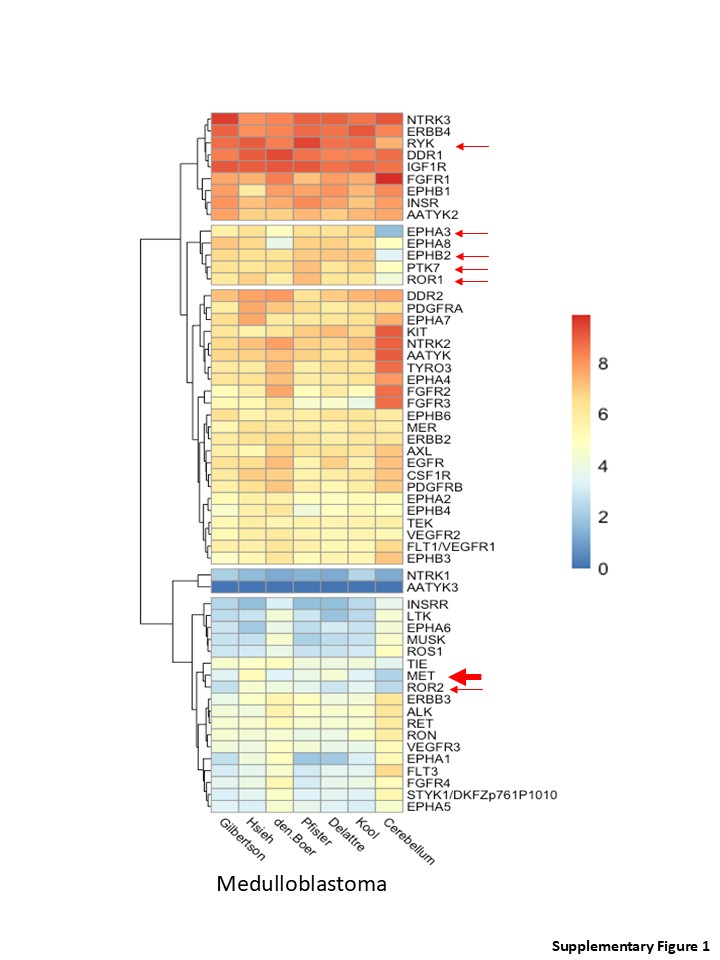

Supplement: Supplementary file 2 — Supplementary Material 2 (Supplementary Fig. 1. Gene expression analysis of tyrosine kinase receptors derived from six different publicly available datasets (Gilbertson n = 76; Hsieh n = 31; denBoer n = 51; Pfister n = 223; Delattre n = 57; Kool n = 62) compared with expression in healthy cerebellum (Roth n = 9). Heatmap representation of gene expression is based on log2 normalised gene expression signal of all analysed RTK. Only seven genes (RYK, ROR1, ROR2, EPHB2, PTK7, EPHA3, MET) out of 29 significantly modulated RTK genes (FLT3, AATYK, FGFR1, TYRO3, KIT, FGFR3, NTRK2, EPHA4, FGFR2, ROS1, RYK, EPHB3, ROR1, EPHB2, STYK1, PTK7, RON, EPHA5, ERBB3, CSF1R, EPHA3, ALK, MET, EPHA7, INSRR, RET, ROR2, PDGFRB, VEGFR3) showed an increased expression in tumour compared to normal (corrected p-value < = 0.05). Heatmap was generated by plotting gene expression values on R software. Red arrows indicate genes significantly overexpressed in MB compared to normal adult cerebella. Tumour samples comprehend a mix of MB specimens belonging to the different subgroups. Supplementary Fig. 2. (A) HGF Log2 normalised expression across six different datasets of medulloblastoma samples compared with healthy cerebella (One way ANOVA p-value = 5.53x10− 16) (Gilbertson n = 76; Hsieh n = 31; den Boer n = 51; Pfister n = 223; Delattre n = 57; Kool n = 62; Roth n = 9). Tumour samples comprehend a mix of MB specimens belonging to the different subgroups. (B) HGF Log2 normalised gene expression in Cavalli dataset (MB samples = 612) across medulloblastoma subgroups (no of samples: WNT = 70; SHH = 223; Group 3 = 144; Group 4 = 326). One way ANOVA p-value = 3.27e-26. (C) HGF Log 2 normalised gene expression across subtypes of medulloblastoma (no of samples: WNT = 21; WNT = 49; SHH = 65; SHH = 35; SHH = 76; SHH = 47; Group 3 = 67; Group 3 = 37; Group 3 = 40; Group 4 = 98; Group 4 = 109; Group 4 = 119). One way ANOVA p-value = 8.77e-34. (D) Correlation between HGF and c-MET expression (from Cavalli et a [file 12672_2026_4717_MOESM2_ESM.zip › Supplementary Figures 1 2 3 4 5 6 7 9 10 11 final/Fig. S1.JPG]

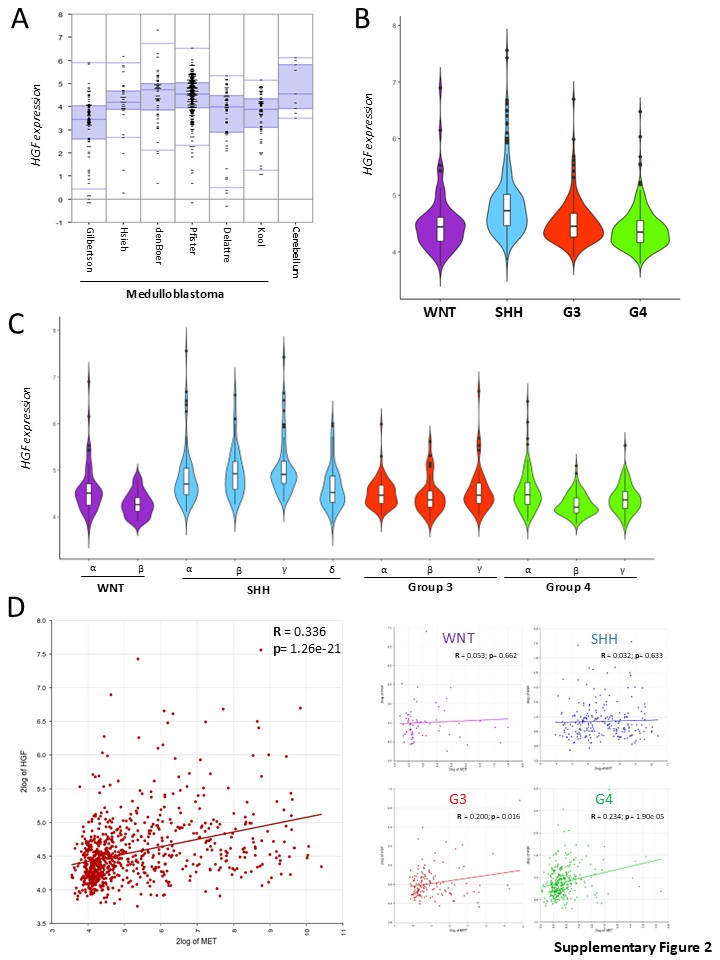

Supplement: Supplementary file 2 — Supplementary Material 2 (Supplementary Fig. 1. Gene expression analysis of tyrosine kinase receptors derived from six different publicly available datasets (Gilbertson n = 76; Hsieh n = 31; denBoer n = 51; Pfister n = 223; Delattre n = 57; Kool n = 62) compared with expression in healthy cerebellum (Roth n = 9). Heatmap representation of gene expression is based on log2 normalised gene expression signal of all analysed RTK. Only seven genes (RYK, ROR1, ROR2, EPHB2, PTK7, EPHA3, MET) out of 29 significantly modulated RTK genes (FLT3, AATYK, FGFR1, TYRO3, KIT, FGFR3, NTRK2, EPHA4, FGFR2, ROS1, RYK, EPHB3, ROR1, EPHB2, STYK1, PTK7, RON, EPHA5, ERBB3, CSF1R, EPHA3, ALK, MET, EPHA7, INSRR, RET, ROR2, PDGFRB, VEGFR3) showed an increased expression in tumour compared to normal (corrected p-value < = 0.05). Heatmap was generated by plotting gene expression values on R software. Red arrows indicate genes significantly overexpressed in MB compared to normal adult cerebella. Tumour samples comprehend a mix of MB specimens belonging to the different subgroups. Supplementary Fig. 2. (A) HGF Log2 normalised expression across six different datasets of medulloblastoma samples compared with healthy cerebella (One way ANOVA p-value = 5.53x10− 16) (Gilbertson n = 76; Hsieh n = 31; den Boer n = 51; Pfister n = 223; Delattre n = 57; Kool n = 62; Roth n = 9). Tumour samples comprehend a mix of MB specimens belonging to the different subgroups. (B) HGF Log2 normalised gene expression in Cavalli dataset (MB samples = 612) across medulloblastoma subgroups (no of samples: WNT = 70; SHH = 223; Group 3 = 144; Group 4 = 326). One way ANOVA p-value = 3.27e-26. (C) HGF Log 2 normalised gene expression across subtypes of medulloblastoma (no of samples: WNT = 21; WNT = 49; SHH = 65; SHH = 35; SHH = 76; SHH = 47; Group 3 = 67; Group 3 = 37; Group 3 = 40; Group 4 = 98; Group 4 = 109; Group 4 = 119). One way ANOVA p-value = 8.77e-34. (D) Correlation between HGF and c-MET expression (from Cavalli et a [file 12672_2026_4717_MOESM2_ESM.zip › Supplementary Figures 1 2 3 4 5 6 7 9 10 11 final/Fig. S2.JPG]

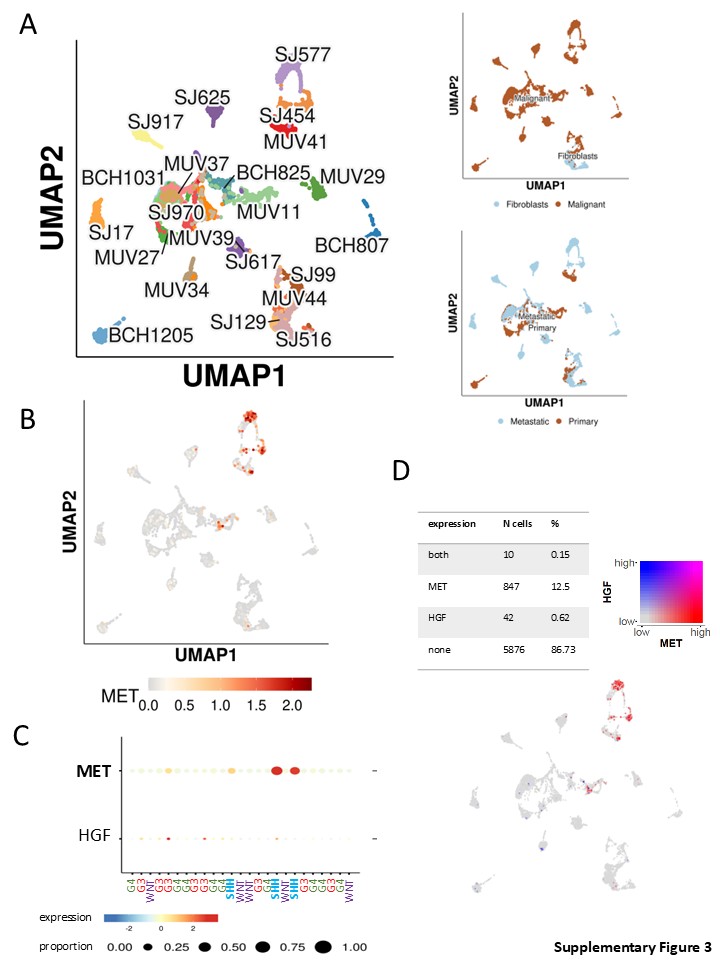

Supplement: Supplementary file 2 — Supplementary Material 2 (Supplementary Fig. 1. Gene expression analysis of tyrosine kinase receptors derived from six different publicly available datasets (Gilbertson n = 76; Hsieh n = 31; denBoer n = 51; Pfister n = 223; Delattre n = 57; Kool n = 62) compared with expression in healthy cerebellum (Roth n = 9). Heatmap representation of gene expression is based on log2 normalised gene expression signal of all analysed RTK. Only seven genes (RYK, ROR1, ROR2, EPHB2, PTK7, EPHA3, MET) out of 29 significantly modulated RTK genes (FLT3, AATYK, FGFR1, TYRO3, KIT, FGFR3, NTRK2, EPHA4, FGFR2, ROS1, RYK, EPHB3, ROR1, EPHB2, STYK1, PTK7, RON, EPHA5, ERBB3, CSF1R, EPHA3, ALK, MET, EPHA7, INSRR, RET, ROR2, PDGFRB, VEGFR3) showed an increased expression in tumour compared to normal (corrected p-value < = 0.05). Heatmap was generated by plotting gene expression values on R software. Red arrows indicate genes significantly overexpressed in MB compared to normal adult cerebella. Tumour samples comprehend a mix of MB specimens belonging to the different subgroups. Supplementary Fig. 2. (A) HGF Log2 normalised expression across six different datasets of medulloblastoma samples compared with healthy cerebella (One way ANOVA p-value = 5.53x10− 16) (Gilbertson n = 76; Hsieh n = 31; den Boer n = 51; Pfister n = 223; Delattre n = 57; Kool n = 62; Roth n = 9). Tumour samples comprehend a mix of MB specimens belonging to the different subgroups. (B) HGF Log2 normalised gene expression in Cavalli dataset (MB samples = 612) across medulloblastoma subgroups (no of samples: WNT = 70; SHH = 223; Group 3 = 144; Group 4 = 326). One way ANOVA p-value = 3.27e-26. (C) HGF Log 2 normalised gene expression across subtypes of medulloblastoma (no of samples: WNT = 21; WNT = 49; SHH = 65; SHH = 35; SHH = 76; SHH = 47; Group 3 = 67; Group 3 = 37; Group 3 = 40; Group 4 = 98; Group 4 = 109; Group 4 = 119). One way ANOVA p-value = 8.77e-34. (D) Correlation between HGF and c-MET expression (from Cavalli et a [file 12672_2026_4717_MOESM2_ESM.zip › Supplementary Figures 1 2 3 4 5 6 7 9 10 11 final/Fig. S3.JPG]

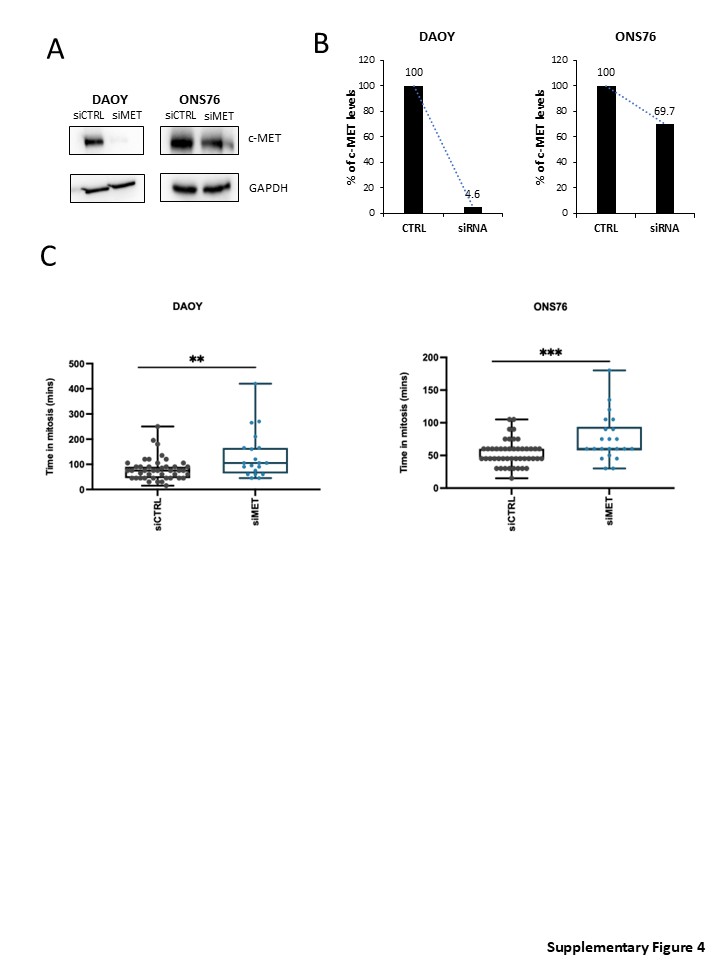

Supplement: Supplementary file 2 — Supplementary Material 2 (Supplementary Fig. 1. Gene expression analysis of tyrosine kinase receptors derived from six different publicly available datasets (Gilbertson n = 76; Hsieh n = 31; denBoer n = 51; Pfister n = 223; Delattre n = 57; Kool n = 62) compared with expression in healthy cerebellum (Roth n = 9). Heatmap representation of gene expression is based on log2 normalised gene expression signal of all analysed RTK. Only seven genes (RYK, ROR1, ROR2, EPHB2, PTK7, EPHA3, MET) out of 29 significantly modulated RTK genes (FLT3, AATYK, FGFR1, TYRO3, KIT, FGFR3, NTRK2, EPHA4, FGFR2, ROS1, RYK, EPHB3, ROR1, EPHB2, STYK1, PTK7, RON, EPHA5, ERBB3, CSF1R, EPHA3, ALK, MET, EPHA7, INSRR, RET, ROR2, PDGFRB, VEGFR3) showed an increased expression in tumour compared to normal (corrected p-value < = 0.05). Heatmap was generated by plotting gene expression values on R software. Red arrows indicate genes significantly overexpressed in MB compared to normal adult cerebella. Tumour samples comprehend a mix of MB specimens belonging to the different subgroups. Supplementary Fig. 2. (A) HGF Log2 normalised expression across six different datasets of medulloblastoma samples compared with healthy cerebella (One way ANOVA p-value = 5.53x10− 16) (Gilbertson n = 76; Hsieh n = 31; den Boer n = 51; Pfister n = 223; Delattre n = 57; Kool n = 62; Roth n = 9). Tumour samples comprehend a mix of MB specimens belonging to the different subgroups. (B) HGF Log2 normalised gene expression in Cavalli dataset (MB samples = 612) across medulloblastoma subgroups (no of samples: WNT = 70; SHH = 223; Group 3 = 144; Group 4 = 326). One way ANOVA p-value = 3.27e-26. (C) HGF Log 2 normalised gene expression across subtypes of medulloblastoma (no of samples: WNT = 21; WNT = 49; SHH = 65; SHH = 35; SHH = 76; SHH = 47; Group 3 = 67; Group 3 = 37; Group 3 = 40; Group 4 = 98; Group 4 = 109; Group 4 = 119). One way ANOVA p-value = 8.77e-34. (D) Correlation between HGF and c-MET expression (from Cavalli et a [file 12672_2026_4717_MOESM2_ESM.zip › Supplementary Figures 1 2 3 4 5 6 7 9 10 11 final/Fig. S4.JPG]

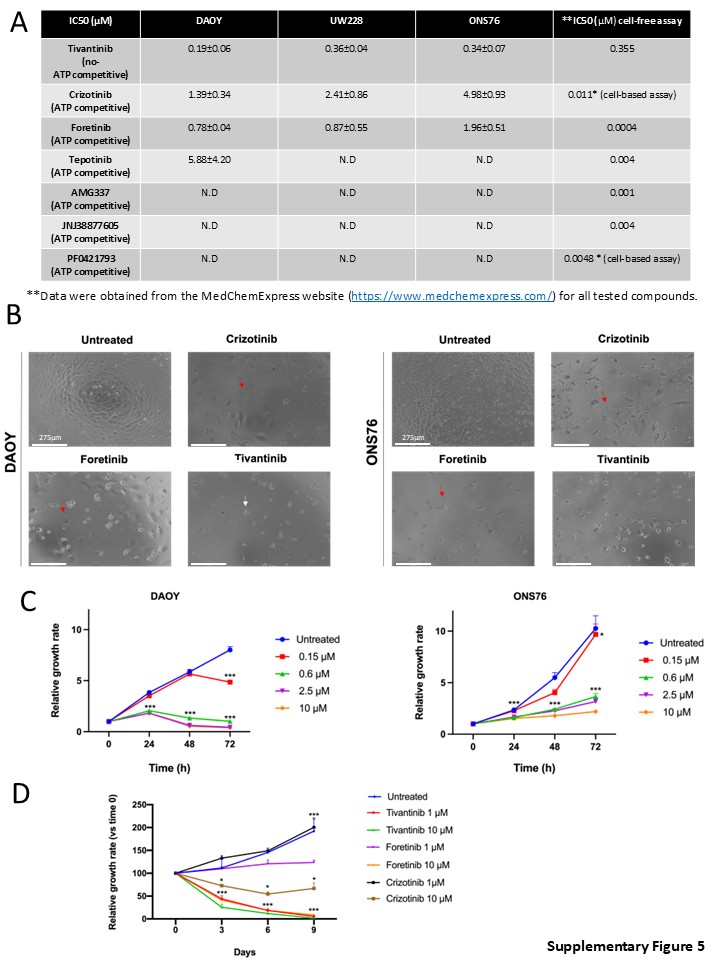

Supplement: Supplementary file 2 — Supplementary Material 2 (Supplementary Fig. 1. Gene expression analysis of tyrosine kinase receptors derived from six different publicly available datasets (Gilbertson n = 76; Hsieh n = 31; denBoer n = 51; Pfister n = 223; Delattre n = 57; Kool n = 62) compared with expression in healthy cerebellum (Roth n = 9). Heatmap representation of gene expression is based on log2 normalised gene expression signal of all analysed RTK. Only seven genes (RYK, ROR1, ROR2, EPHB2, PTK7, EPHA3, MET) out of 29 significantly modulated RTK genes (FLT3, AATYK, FGFR1, TYRO3, KIT, FGFR3, NTRK2, EPHA4, FGFR2, ROS1, RYK, EPHB3, ROR1, EPHB2, STYK1, PTK7, RON, EPHA5, ERBB3, CSF1R, EPHA3, ALK, MET, EPHA7, INSRR, RET, ROR2, PDGFRB, VEGFR3) showed an increased expression in tumour compared to normal (corrected p-value < = 0.05). Heatmap was generated by plotting gene expression values on R software. Red arrows indicate genes significantly overexpressed in MB compared to normal adult cerebella. Tumour samples comprehend a mix of MB specimens belonging to the different subgroups. Supplementary Fig. 2. (A) HGF Log2 normalised expression across six different datasets of medulloblastoma samples compared with healthy cerebella (One way ANOVA p-value = 5.53x10− 16) (Gilbertson n = 76; Hsieh n = 31; den Boer n = 51; Pfister n = 223; Delattre n = 57; Kool n = 62; Roth n = 9). Tumour samples comprehend a mix of MB specimens belonging to the different subgroups. (B) HGF Log2 normalised gene expression in Cavalli dataset (MB samples = 612) across medulloblastoma subgroups (no of samples: WNT = 70; SHH = 223; Group 3 = 144; Group 4 = 326). One way ANOVA p-value = 3.27e-26. (C) HGF Log 2 normalised gene expression across subtypes of medulloblastoma (no of samples: WNT = 21; WNT = 49; SHH = 65; SHH = 35; SHH = 76; SHH = 47; Group 3 = 67; Group 3 = 37; Group 3 = 40; Group 4 = 98; Group 4 = 109; Group 4 = 119). One way ANOVA p-value = 8.77e-34. (D) Correlation between HGF and c-MET expression (from Cavalli et a [file 12672_2026_4717_MOESM2_ESM.zip › Supplementary Figures 1 2 3 4 5 6 7 9 10 11 final/Fig. S5.JPG]

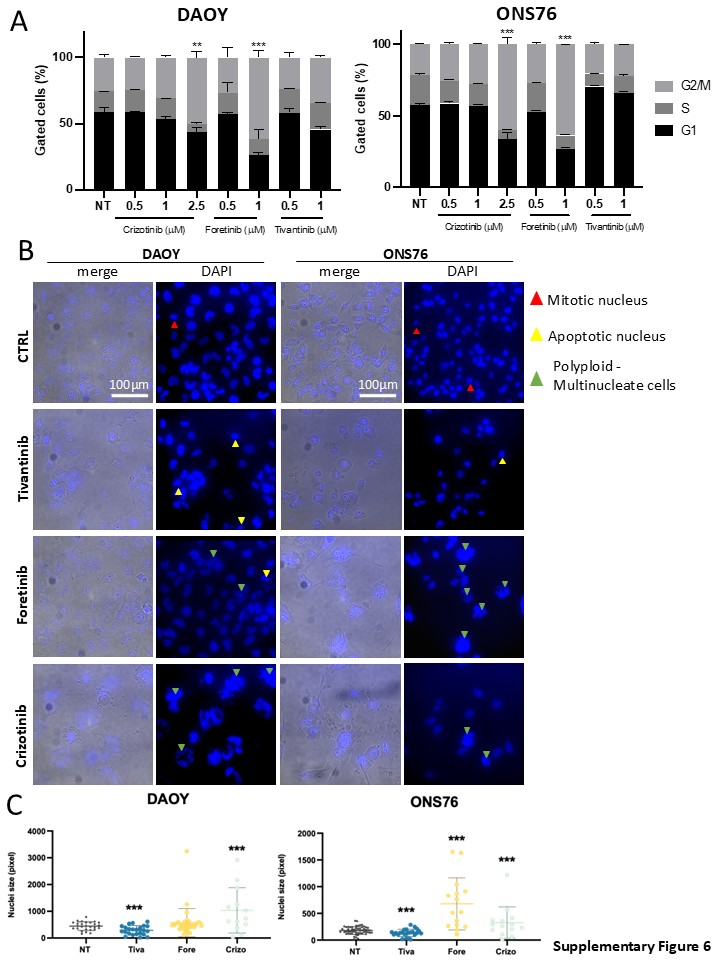

Supplement: Supplementary file 2 — Supplementary Material 2 (Supplementary Fig. 1. Gene expression analysis of tyrosine kinase receptors derived from six different publicly available datasets (Gilbertson n = 76; Hsieh n = 31; denBoer n = 51; Pfister n = 223; Delattre n = 57; Kool n = 62) compared with expression in healthy cerebellum (Roth n = 9). Heatmap representation of gene expression is based on log2 normalised gene expression signal of all analysed RTK. Only seven genes (RYK, ROR1, ROR2, EPHB2, PTK7, EPHA3, MET) out of 29 significantly modulated RTK genes (FLT3, AATYK, FGFR1, TYRO3, KIT, FGFR3, NTRK2, EPHA4, FGFR2, ROS1, RYK, EPHB3, ROR1, EPHB2, STYK1, PTK7, RON, EPHA5, ERBB3, CSF1R, EPHA3, ALK, MET, EPHA7, INSRR, RET, ROR2, PDGFRB, VEGFR3) showed an increased expression in tumour compared to normal (corrected p-value < = 0.05). Heatmap was generated by plotting gene expression values on R software. Red arrows indicate genes significantly overexpressed in MB compared to normal adult cerebella. Tumour samples comprehend a mix of MB specimens belonging to the different subgroups. Supplementary Fig. 2. (A) HGF Log2 normalised expression across six different datasets of medulloblastoma samples compared with healthy cerebella (One way ANOVA p-value = 5.53x10− 16) (Gilbertson n = 76; Hsieh n = 31; den Boer n = 51; Pfister n = 223; Delattre n = 57; Kool n = 62; Roth n = 9). Tumour samples comprehend a mix of MB specimens belonging to the different subgroups. (B) HGF Log2 normalised gene expression in Cavalli dataset (MB samples = 612) across medulloblastoma subgroups (no of samples: WNT = 70; SHH = 223; Group 3 = 144; Group 4 = 326). One way ANOVA p-value = 3.27e-26. (C) HGF Log 2 normalised gene expression across subtypes of medulloblastoma (no of samples: WNT = 21; WNT = 49; SHH = 65; SHH = 35; SHH = 76; SHH = 47; Group 3 = 67; Group 3 = 37; Group 3 = 40; Group 4 = 98; Group 4 = 109; Group 4 = 119). One way ANOVA p-value = 8.77e-34. (D) Correlation between HGF and c-MET expression (from Cavalli et a [file 12672_2026_4717_MOESM2_ESM.zip › Supplementary Figures 1 2 3 4 5 6 7 9 10 11 final/Fig. S6.JPG]

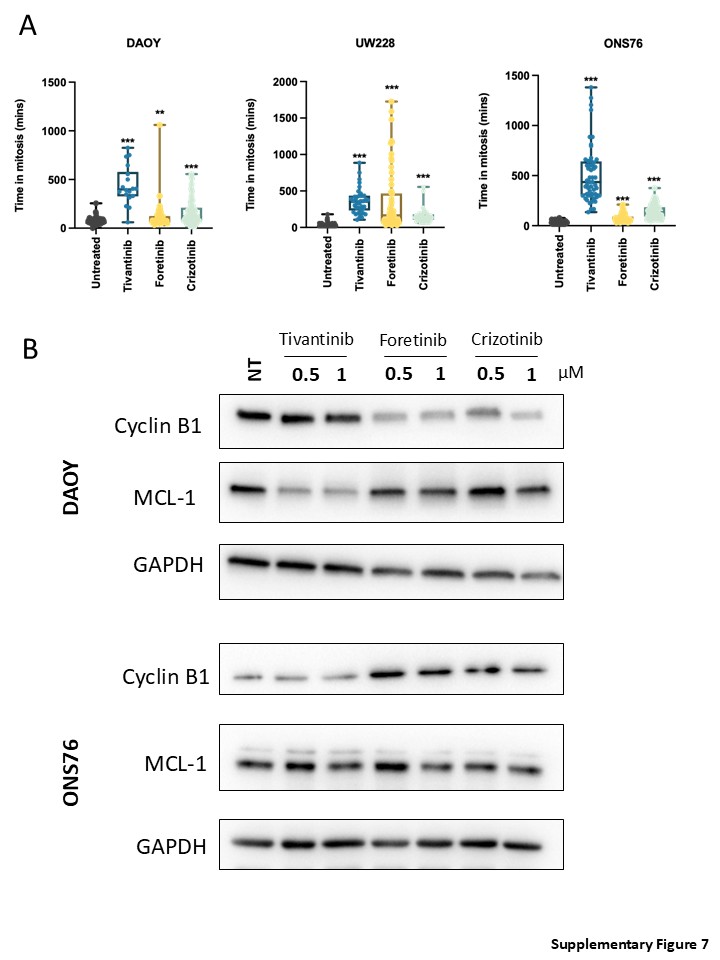

Supplement: Supplementary file 2 — Supplementary Material 2 (Supplementary Fig. 1. Gene expression analysis of tyrosine kinase receptors derived from six different publicly available datasets (Gilbertson n = 76; Hsieh n = 31; denBoer n = 51; Pfister n = 223; Delattre n = 57; Kool n = 62) compared with expression in healthy cerebellum (Roth n = 9). Heatmap representation of gene expression is based on log2 normalised gene expression signal of all analysed RTK. Only seven genes (RYK, ROR1, ROR2, EPHB2, PTK7, EPHA3, MET) out of 29 significantly modulated RTK genes (FLT3, AATYK, FGFR1, TYRO3, KIT, FGFR3, NTRK2, EPHA4, FGFR2, ROS1, RYK, EPHB3, ROR1, EPHB2, STYK1, PTK7, RON, EPHA5, ERBB3, CSF1R, EPHA3, ALK, MET, EPHA7, INSRR, RET, ROR2, PDGFRB, VEGFR3) showed an increased expression in tumour compared to normal (corrected p-value < = 0.05). Heatmap was generated by plotting gene expression values on R software. Red arrows indicate genes significantly overexpressed in MB compared to normal adult cerebella. Tumour samples comprehend a mix of MB specimens belonging to the different subgroups. Supplementary Fig. 2. (A) HGF Log2 normalised expression across six different datasets of medulloblastoma samples compared with healthy cerebella (One way ANOVA p-value = 5.53x10− 16) (Gilbertson n = 76; Hsieh n = 31; den Boer n = 51; Pfister n = 223; Delattre n = 57; Kool n = 62; Roth n = 9). Tumour samples comprehend a mix of MB specimens belonging to the different subgroups. (B) HGF Log2 normalised gene expression in Cavalli dataset (MB samples = 612) across medulloblastoma subgroups (no of samples: WNT = 70; SHH = 223; Group 3 = 144; Group 4 = 326). One way ANOVA p-value = 3.27e-26. (C) HGF Log 2 normalised gene expression across subtypes of medulloblastoma (no of samples: WNT = 21; WNT = 49; SHH = 65; SHH = 35; SHH = 76; SHH = 47; Group 3 = 67; Group 3 = 37; Group 3 = 40; Group 4 = 98; Group 4 = 109; Group 4 = 119). One way ANOVA p-value = 8.77e-34. (D) Correlation between HGF and c-MET expression (from Cavalli et a [file 12672_2026_4717_MOESM2_ESM.zip › Supplementary Figures 1 2 3 4 5 6 7 9 10 11 final/Fig. S7.JPG]

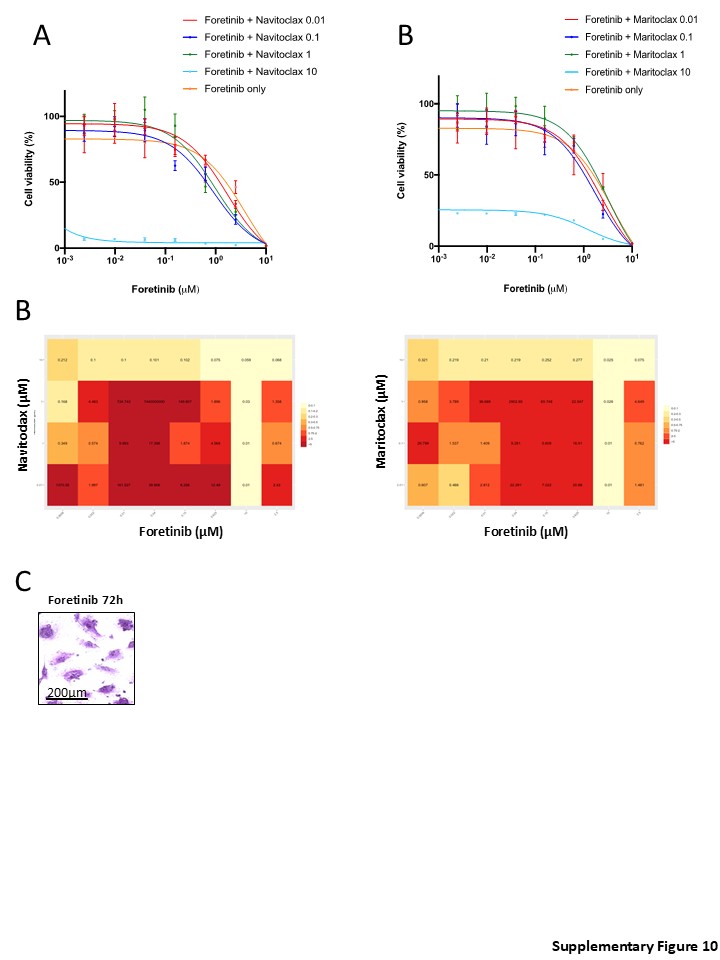

Supplement: Supplementary file 2 — Supplementary Material 2 (Supplementary Fig. 1. Gene expression analysis of tyrosine kinase receptors derived from six different publicly available datasets (Gilbertson n = 76; Hsieh n = 31; denBoer n = 51; Pfister n = 223; Delattre n = 57; Kool n = 62) compared with expression in healthy cerebellum (Roth n = 9). Heatmap representation of gene expression is based on log2 normalised gene expression signal of all analysed RTK. Only seven genes (RYK, ROR1, ROR2, EPHB2, PTK7, EPHA3, MET) out of 29 significantly modulated RTK genes (FLT3, AATYK, FGFR1, TYRO3, KIT, FGFR3, NTRK2, EPHA4, FGFR2, ROS1, RYK, EPHB3, ROR1, EPHB2, STYK1, PTK7, RON, EPHA5, ERBB3, CSF1R, EPHA3, ALK, MET, EPHA7, INSRR, RET, ROR2, PDGFRB, VEGFR3) showed an increased expression in tumour compared to normal (corrected p-value < = 0.05). Heatmap was generated by plotting gene expression values on R software. Red arrows indicate genes significantly overexpressed in MB compared to normal adult cerebella. Tumour samples comprehend a mix of MB specimens belonging to the different subgroups. Supplementary Fig. 2. (A) HGF Log2 normalised expression across six different datasets of medulloblastoma samples compared with healthy cerebella (One way ANOVA p-value = 5.53x10− 16) (Gilbertson n = 76; Hsieh n = 31; den Boer n = 51; Pfister n = 223; Delattre n = 57; Kool n = 62; Roth n = 9). Tumour samples comprehend a mix of MB specimens belonging to the different subgroups. (B) HGF Log2 normalised gene expression in Cavalli dataset (MB samples = 612) across medulloblastoma subgroups (no of samples: WNT = 70; SHH = 223; Group 3 = 144; Group 4 = 326). One way ANOVA p-value = 3.27e-26. (C) HGF Log 2 normalised gene expression across subtypes of medulloblastoma (no of samples: WNT = 21; WNT = 49; SHH = 65; SHH = 35; SHH = 76; SHH = 47; Group 3 = 67; Group 3 = 37; Group 3 = 40; Group 4 = 98; Group 4 = 109; Group 4 = 119). One way ANOVA p-value = 8.77e-34. (D) Correlation between HGF and c-MET expression (from Cavalli et a [file 12672_2026_4717_MOESM2_ESM.zip › Supplementary Figures 1 2 3 4 5 6 7 9 10 11 final/Fig. S10.JPG]

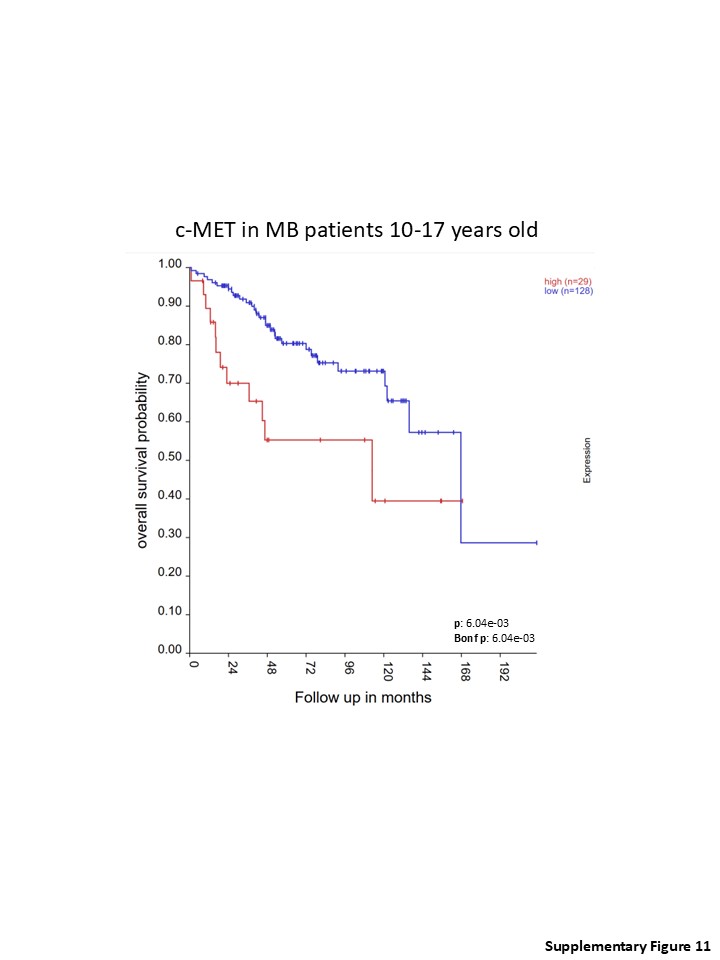

Supplement: Supplementary file 2 — Supplementary Material 2 (Supplementary Fig. 1. Gene expression analysis of tyrosine kinase receptors derived from six different publicly available datasets (Gilbertson n = 76; Hsieh n = 31; denBoer n = 51; Pfister n = 223; Delattre n = 57; Kool n = 62) compared with expression in healthy cerebellum (Roth n = 9). Heatmap representation of gene expression is based on log2 normalised gene expression signal of all analysed RTK. Only seven genes (RYK, ROR1, ROR2, EPHB2, PTK7, EPHA3, MET) out of 29 significantly modulated RTK genes (FLT3, AATYK, FGFR1, TYRO3, KIT, FGFR3, NTRK2, EPHA4, FGFR2, ROS1, RYK, EPHB3, ROR1, EPHB2, STYK1, PTK7, RON, EPHA5, ERBB3, CSF1R, EPHA3, ALK, MET, EPHA7, INSRR, RET, ROR2, PDGFRB, VEGFR3) showed an increased expression in tumour compared to normal (corrected p-value < = 0.05). Heatmap was generated by plotting gene expression values on R software. Red arrows indicate genes significantly overexpressed in MB compared to normal adult cerebella. Tumour samples comprehend a mix of MB specimens belonging to the different subgroups. Supplementary Fig. 2. (A) HGF Log2 normalised expression across six different datasets of medulloblastoma samples compared with healthy cerebella (One way ANOVA p-value = 5.53x10− 16) (Gilbertson n = 76; Hsieh n = 31; den Boer n = 51; Pfister n = 223; Delattre n = 57; Kool n = 62; Roth n = 9). Tumour samples comprehend a mix of MB specimens belonging to the different subgroups. (B) HGF Log2 normalised gene expression in Cavalli dataset (MB samples = 612) across medulloblastoma subgroups (no of samples: WNT = 70; SHH = 223; Group 3 = 144; Group 4 = 326). One way ANOVA p-value = 3.27e-26. (C) HGF Log 2 normalised gene expression across subtypes of medulloblastoma (no of samples: WNT = 21; WNT = 49; SHH = 65; SHH = 35; SHH = 76; SHH = 47; Group 3 = 67; Group 3 = 37; Group 3 = 40; Group 4 = 98; Group 4 = 109; Group 4 = 119). One way ANOVA p-value = 8.77e-34. (D) Correlation between HGF and c-MET expression (from Cavalli et a [file 12672_2026_4717_MOESM2_ESM.zip › Supplementary Figures 1 2 3 4 5 6 7 9 10 11 final/Fig. S11.JPG]

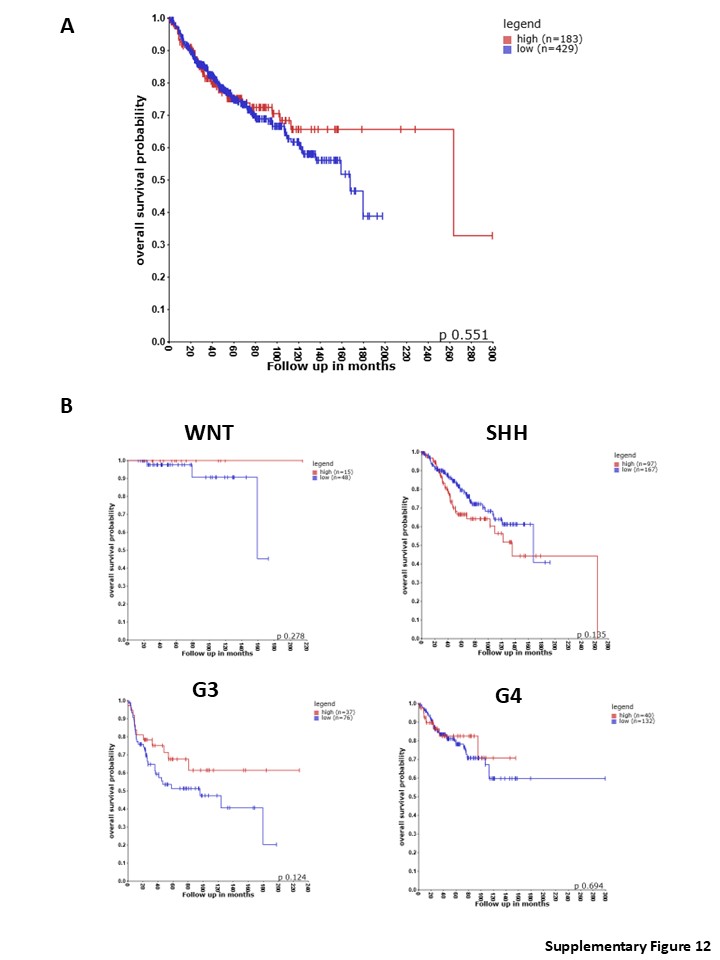

Supplement: Supplementary file 2 — Supplementary Material 2 (Supplementary Fig. 1. Gene expression analysis of tyrosine kinase receptors derived from six different publicly available datasets (Gilbertson n = 76; Hsieh n = 31; denBoer n = 51; Pfister n = 223; Delattre n = 57; Kool n = 62) compared with expression in healthy cerebellum (Roth n = 9). Heatmap representation of gene expression is based on log2 normalised gene expression signal of all analysed RTK. Only seven genes (RYK, ROR1, ROR2, EPHB2, PTK7, EPHA3, MET) out of 29 significantly modulated RTK genes (FLT3, AATYK, FGFR1, TYRO3, KIT, FGFR3, NTRK2, EPHA4, FGFR2, ROS1, RYK, EPHB3, ROR1, EPHB2, STYK1, PTK7, RON, EPHA5, ERBB3, CSF1R, EPHA3, ALK, MET, EPHA7, INSRR, RET, ROR2, PDGFRB, VEGFR3) showed an increased expression in tumour compared to normal (corrected p-value < = 0.05). Heatmap was generated by plotting gene expression values on R software. Red arrows indicate genes significantly overexpressed in MB compared to normal adult cerebella. Tumour samples comprehend a mix of MB specimens belonging to the different subgroups. Supplementary Fig. 2. (A) HGF Log2 normalised expression across six different datasets of medulloblastoma samples compared with healthy cerebella (One way ANOVA p-value = 5.53x10− 16) (Gilbertson n = 76; Hsieh n = 31; den Boer n = 51; Pfister n = 223; Delattre n = 57; Kool n = 62; Roth n = 9). Tumour samples comprehend a mix of MB specimens belonging to the different subgroups. (B) HGF Log2 normalised gene expression in Cavalli dataset (MB samples = 612) across medulloblastoma subgroups (no of samples: WNT = 70; SHH = 223; Group 3 = 144; Group 4 = 326). One way ANOVA p-value = 3.27e-26. (C) HGF Log 2 normalised gene expression across subtypes of medulloblastoma (no of samples: WNT = 21; WNT = 49; SHH = 65; SHH = 35; SHH = 76; SHH = 47; Group 3 = 67; Group 3 = 37; Group 3 = 40; Group 4 = 98; Group 4 = 109; Group 4 = 119). One way ANOVA p-value = 8.77e-34. (D) Correlation between HGF and c-MET expression (from Cavalli et a [file 12672_2026_4717_MOESM2_ESM.zip › Supplementary Figures 1 2 3 4 5 6 7 9 10 11 final/Fig. S12.JPG]

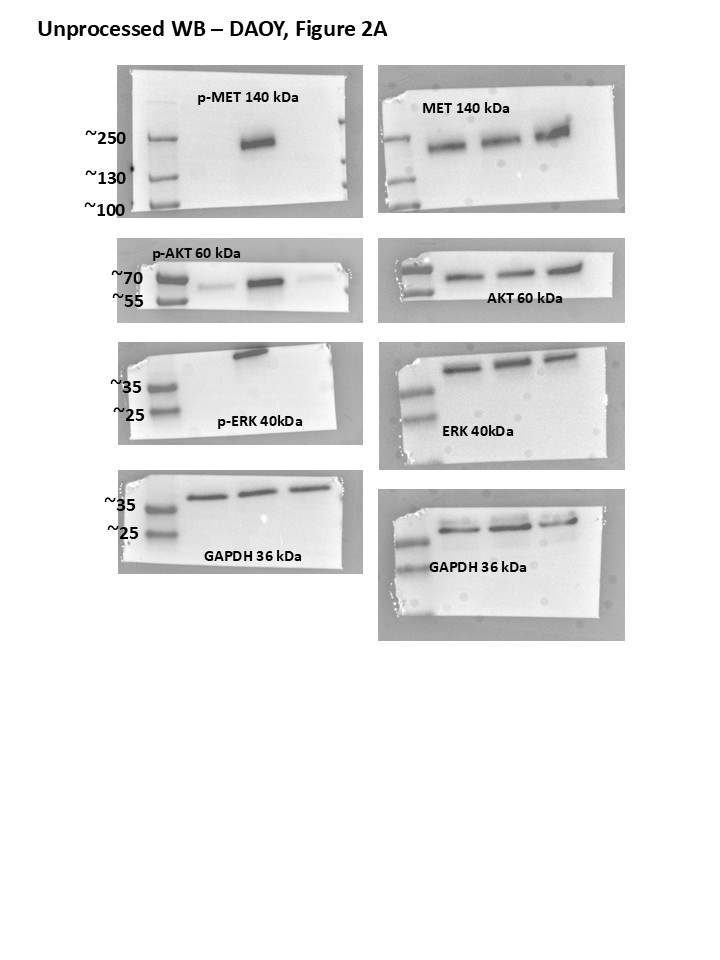

Supplement: Supplementary file 2 — Supplementary Material 2 (Supplementary Fig. 1. Gene expression analysis of tyrosine kinase receptors derived from six different publicly available datasets (Gilbertson n = 76; Hsieh n = 31; denBoer n = 51; Pfister n = 223; Delattre n = 57; Kool n = 62) compared with expression in healthy cerebellum (Roth n = 9). Heatmap representation of gene expression is based on log2 normalised gene expression signal of all analysed RTK. Only seven genes (RYK, ROR1, ROR2, EPHB2, PTK7, EPHA3, MET) out of 29 significantly modulated RTK genes (FLT3, AATYK, FGFR1, TYRO3, KIT, FGFR3, NTRK2, EPHA4, FGFR2, ROS1, RYK, EPHB3, ROR1, EPHB2, STYK1, PTK7, RON, EPHA5, ERBB3, CSF1R, EPHA3, ALK, MET, EPHA7, INSRR, RET, ROR2, PDGFRB, VEGFR3) showed an increased expression in tumour compared to normal (corrected p-value < = 0.05). Heatmap was generated by plotting gene expression values on R software. Red arrows indicate genes significantly overexpressed in MB compared to normal adult cerebella. Tumour samples comprehend a mix of MB specimens belonging to the different subgroups. Supplementary Fig. 2. (A) HGF Log2 normalised expression across six different datasets of medulloblastoma samples compared with healthy cerebella (One way ANOVA p-value = 5.53x10− 16) (Gilbertson n = 76; Hsieh n = 31; den Boer n = 51; Pfister n = 223; Delattre n = 57; Kool n = 62; Roth n = 9). Tumour samples comprehend a mix of MB specimens belonging to the different subgroups. (B) HGF Log2 normalised gene expression in Cavalli dataset (MB samples = 612) across medulloblastoma subgroups (no of samples: WNT = 70; SHH = 223; Group 3 = 144; Group 4 = 326). One way ANOVA p-value = 3.27e-26. (C) HGF Log 2 normalised gene expression across subtypes of medulloblastoma (no of samples: WNT = 21; WNT = 49; SHH = 65; SHH = 35; SHH = 76; SHH = 47; Group 3 = 67; Group 3 = 37; Group 3 = 40; Group 4 = 98; Group 4 = 109; Group 4 = 119). One way ANOVA p-value = 8.77e-34. (D) Correlation between HGF and c-MET expression (from Cavalli et a [file 12672_2026_4717_MOESM2_ESM.zip › Supplementary Figures 1 2 3 4 5 6 7 9 10 11 final/Slide11.JPG]

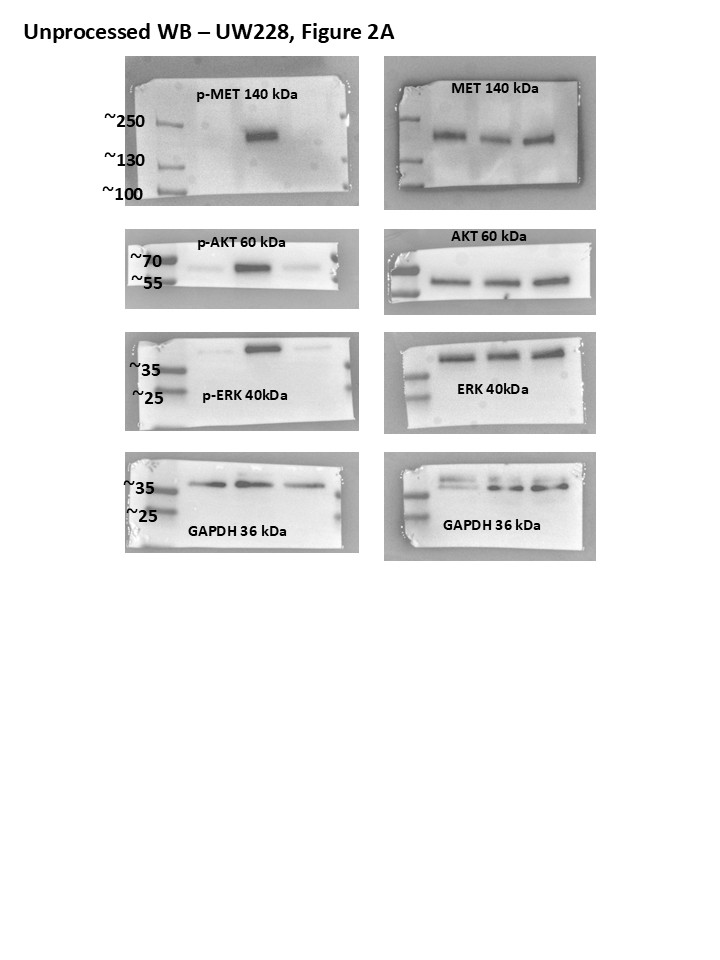

Supplement: Supplementary file 2 — Supplementary Material 2 (Supplementary Fig. 1. Gene expression analysis of tyrosine kinase receptors derived from six different publicly available datasets (Gilbertson n = 76; Hsieh n = 31; denBoer n = 51; Pfister n = 223; Delattre n = 57; Kool n = 62) compared with expression in healthy cerebellum (Roth n = 9). Heatmap representation of gene expression is based on log2 normalised gene expression signal of all analysed RTK. Only seven genes (RYK, ROR1, ROR2, EPHB2, PTK7, EPHA3, MET) out of 29 significantly modulated RTK genes (FLT3, AATYK, FGFR1, TYRO3, KIT, FGFR3, NTRK2, EPHA4, FGFR2, ROS1, RYK, EPHB3, ROR1, EPHB2, STYK1, PTK7, RON, EPHA5, ERBB3, CSF1R, EPHA3, ALK, MET, EPHA7, INSRR, RET, ROR2, PDGFRB, VEGFR3) showed an increased expression in tumour compared to normal (corrected p-value < = 0.05). Heatmap was generated by plotting gene expression values on R software. Red arrows indicate genes significantly overexpressed in MB compared to normal adult cerebella. Tumour samples comprehend a mix of MB specimens belonging to the different subgroups. Supplementary Fig. 2. (A) HGF Log2 normalised expression across six different datasets of medulloblastoma samples compared with healthy cerebella (One way ANOVA p-value = 5.53x10− 16) (Gilbertson n = 76; Hsieh n = 31; den Boer n = 51; Pfister n = 223; Delattre n = 57; Kool n = 62; Roth n = 9). Tumour samples comprehend a mix of MB specimens belonging to the different subgroups. (B) HGF Log2 normalised gene expression in Cavalli dataset (MB samples = 612) across medulloblastoma subgroups (no of samples: WNT = 70; SHH = 223; Group 3 = 144; Group 4 = 326). One way ANOVA p-value = 3.27e-26. (C) HGF Log 2 normalised gene expression across subtypes of medulloblastoma (no of samples: WNT = 21; WNT = 49; SHH = 65; SHH = 35; SHH = 76; SHH = 47; Group 3 = 67; Group 3 = 37; Group 3 = 40; Group 4 = 98; Group 4 = 109; Group 4 = 119). One way ANOVA p-value = 8.77e-34. (D) Correlation between HGF and c-MET expression (from Cavalli et a [file 12672_2026_4717_MOESM2_ESM.zip › Supplementary Figures 1 2 3 4 5 6 7 9 10 11 final/Slide12.JPG]

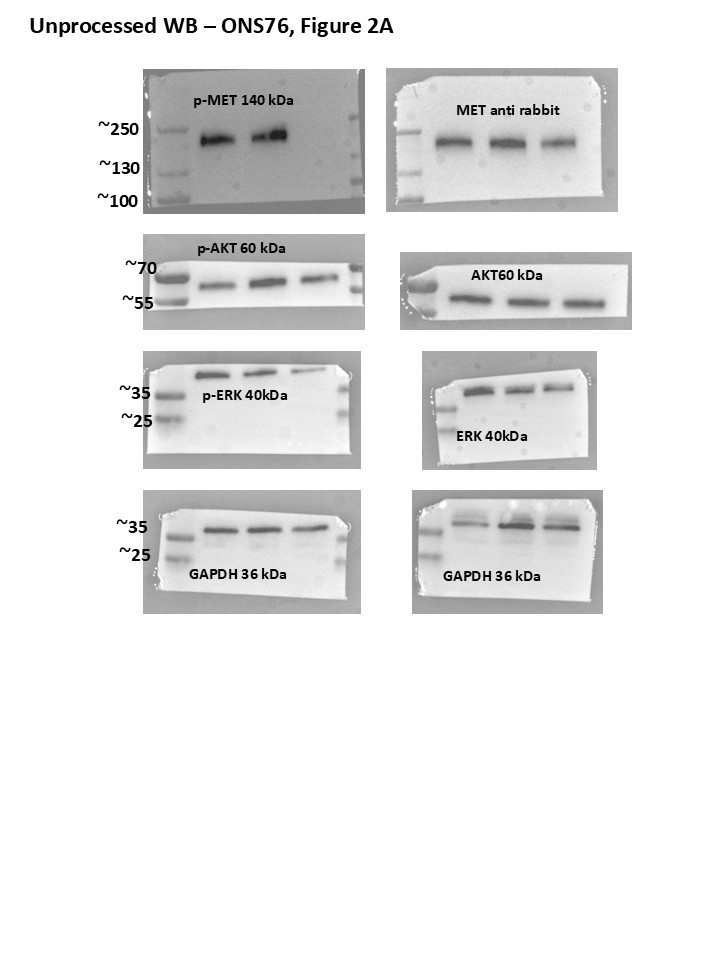

Supplement: Supplementary file 2 — Supplementary Material 2 (Supplementary Fig. 1. Gene expression analysis of tyrosine kinase receptors derived from six different publicly available datasets (Gilbertson n = 76; Hsieh n = 31; denBoer n = 51; Pfister n = 223; Delattre n = 57; Kool n = 62) compared with expression in healthy cerebellum (Roth n = 9). Heatmap representation of gene expression is based on log2 normalised gene expression signal of all analysed RTK. Only seven genes (RYK, ROR1, ROR2, EPHB2, PTK7, EPHA3, MET) out of 29 significantly modulated RTK genes (FLT3, AATYK, FGFR1, TYRO3, KIT, FGFR3, NTRK2, EPHA4, FGFR2, ROS1, RYK, EPHB3, ROR1, EPHB2, STYK1, PTK7, RON, EPHA5, ERBB3, CSF1R, EPHA3, ALK, MET, EPHA7, INSRR, RET, ROR2, PDGFRB, VEGFR3) showed an increased expression in tumour compared to normal (corrected p-value < = 0.05). Heatmap was generated by plotting gene expression values on R software. Red arrows indicate genes significantly overexpressed in MB compared to normal adult cerebella. Tumour samples comprehend a mix of MB specimens belonging to the different subgroups. Supplementary Fig. 2. (A) HGF Log2 normalised expression across six different datasets of medulloblastoma samples compared with healthy cerebella (One way ANOVA p-value = 5.53x10− 16) (Gilbertson n = 76; Hsieh n = 31; den Boer n = 51; Pfister n = 223; Delattre n = 57; Kool n = 62; Roth n = 9). Tumour samples comprehend a mix of MB specimens belonging to the different subgroups. (B) HGF Log2 normalised gene expression in Cavalli dataset (MB samples = 612) across medulloblastoma subgroups (no of samples: WNT = 70; SHH = 223; Group 3 = 144; Group 4 = 326). One way ANOVA p-value = 3.27e-26. (C) HGF Log 2 normalised gene expression across subtypes of medulloblastoma (no of samples: WNT = 21; WNT = 49; SHH = 65; SHH = 35; SHH = 76; SHH = 47; Group 3 = 67; Group 3 = 37; Group 3 = 40; Group 4 = 98; Group 4 = 109; Group 4 = 119). One way ANOVA p-value = 8.77e-34. (D) Correlation between HGF and c-MET expression (from Cavalli et a [file 12672_2026_4717_MOESM2_ESM.zip › Supplementary Figures 1 2 3 4 5 6 7 9 10 11 final/Slide13.JPG]

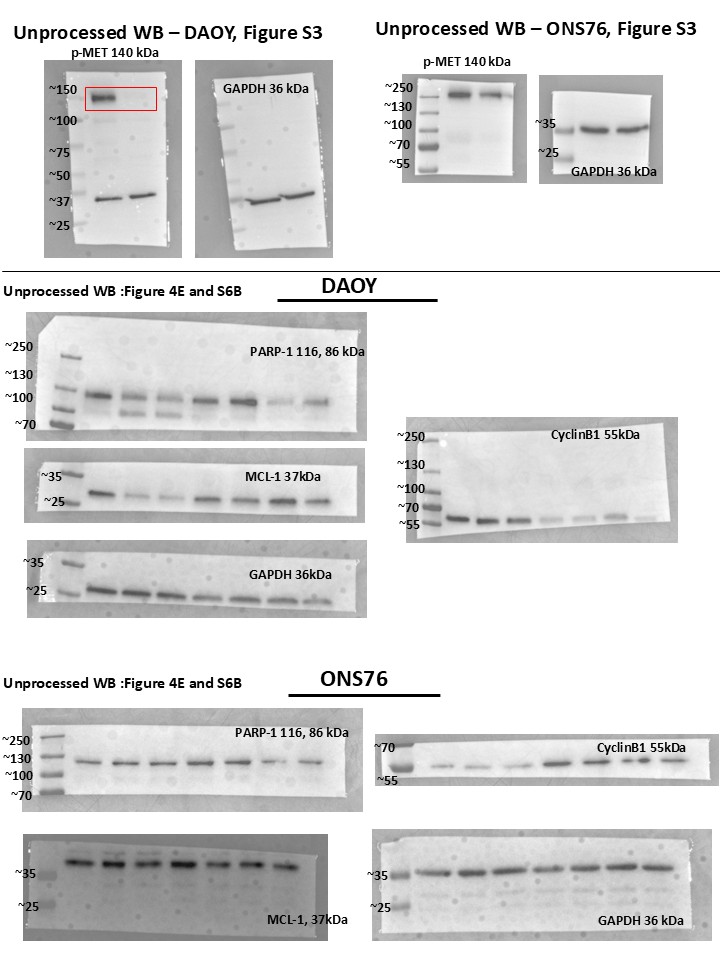

Supplement: Supplementary file 2 — Supplementary Material 2 (Supplementary Fig. 1. Gene expression analysis of tyrosine kinase receptors derived from six different publicly available datasets (Gilbertson n = 76; Hsieh n = 31; denBoer n = 51; Pfister n = 223; Delattre n = 57; Kool n = 62) compared with expression in healthy cerebellum (Roth n = 9). Heatmap representation of gene expression is based on log2 normalised gene expression signal of all analysed RTK. Only seven genes (RYK, ROR1, ROR2, EPHB2, PTK7, EPHA3, MET) out of 29 significantly modulated RTK genes (FLT3, AATYK, FGFR1, TYRO3, KIT, FGFR3, NTRK2, EPHA4, FGFR2, ROS1, RYK, EPHB3, ROR1, EPHB2, STYK1, PTK7, RON, EPHA5, ERBB3, CSF1R, EPHA3, ALK, MET, EPHA7, INSRR, RET, ROR2, PDGFRB, VEGFR3) showed an increased expression in tumour compared to normal (corrected p-value < = 0.05). Heatmap was generated by plotting gene expression values on R software. Red arrows indicate genes significantly overexpressed in MB compared to normal adult cerebella. Tumour samples comprehend a mix of MB specimens belonging to the different subgroups. Supplementary Fig. 2. (A) HGF Log2 normalised expression across six different datasets of medulloblastoma samples compared with healthy cerebella (One way ANOVA p-value = 5.53x10− 16) (Gilbertson n = 76; Hsieh n = 31; den Boer n = 51; Pfister n = 223; Delattre n = 57; Kool n = 62; Roth n = 9). Tumour samples comprehend a mix of MB specimens belonging to the different subgroups. (B) HGF Log2 normalised gene expression in Cavalli dataset (MB samples = 612) across medulloblastoma subgroups (no of samples: WNT = 70; SHH = 223; Group 3 = 144; Group 4 = 326). One way ANOVA p-value = 3.27e-26. (C) HGF Log 2 normalised gene expression across subtypes of medulloblastoma (no of samples: WNT = 21; WNT = 49; SHH = 65; SHH = 35; SHH = 76; SHH = 47; Group 3 = 67; Group 3 = 37; Group 3 = 40; Group 4 = 98; Group 4 = 109; Group 4 = 119). One way ANOVA p-value = 8.77e-34. (D) Correlation between HGF and c-MET expression (from Cavalli et a [file 12672_2026_4717_MOESM2_ESM.zip › Supplementary Figures 1 2 3 4 5 6 7 9 10 11 final/Slide14.JPG]

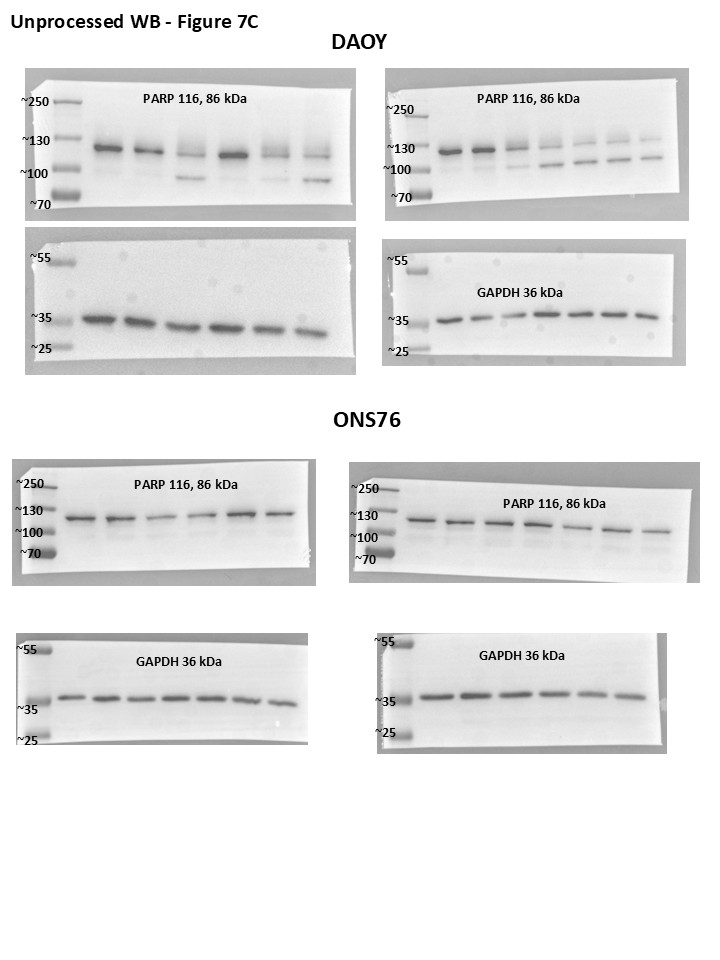

Supplement: Supplementary file 2 — Supplementary Material 2 (Supplementary Fig. 1. Gene expression analysis of tyrosine kinase receptors derived from six different publicly available datasets (Gilbertson n = 76; Hsieh n = 31; denBoer n = 51; Pfister n = 223; Delattre n = 57; Kool n = 62) compared with expression in healthy cerebellum (Roth n = 9). Heatmap representation of gene expression is based on log2 normalised gene expression signal of all analysed RTK. Only seven genes (RYK, ROR1, ROR2, EPHB2, PTK7, EPHA3, MET) out of 29 significantly modulated RTK genes (FLT3, AATYK, FGFR1, TYRO3, KIT, FGFR3, NTRK2, EPHA4, FGFR2, ROS1, RYK, EPHB3, ROR1, EPHB2, STYK1, PTK7, RON, EPHA5, ERBB3, CSF1R, EPHA3, ALK, MET, EPHA7, INSRR, RET, ROR2, PDGFRB, VEGFR3) showed an increased expression in tumour compared to normal (corrected p-value < = 0.05). Heatmap was generated by plotting gene expression values on R software. Red arrows indicate genes significantly overexpressed in MB compared to normal adult cerebella. Tumour samples comprehend a mix of MB specimens belonging to the different subgroups. Supplementary Fig. 2. (A) HGF Log2 normalised expression across six different datasets of medulloblastoma samples compared with healthy cerebella (One way ANOVA p-value = 5.53x10− 16) (Gilbertson n = 76; Hsieh n = 31; den Boer n = 51; Pfister n = 223; Delattre n = 57; Kool n = 62; Roth n = 9). Tumour samples comprehend a mix of MB specimens belonging to the different subgroups. (B) HGF Log2 normalised gene expression in Cavalli dataset (MB samples = 612) across medulloblastoma subgroups (no of samples: WNT = 70; SHH = 223; Group 3 = 144; Group 4 = 326). One way ANOVA p-value = 3.27e-26. (C) HGF Log 2 normalised gene expression across subtypes of medulloblastoma (no of samples: WNT = 21; WNT = 49; SHH = 65; SHH = 35; SHH = 76; SHH = 47; Group 3 = 67; Group 3 = 37; Group 3 = 40; Group 4 = 98; Group 4 = 109; Group 4 = 119). One way ANOVA p-value = 8.77e-34. (D) Correlation between HGF and c-MET expression (from Cavalli et a [file 12672_2026_4717_MOESM2_ESM.zip › Supplementary Figures 1 2 3 4 5 6 7 9 10 11 final/Slide15.JPG]

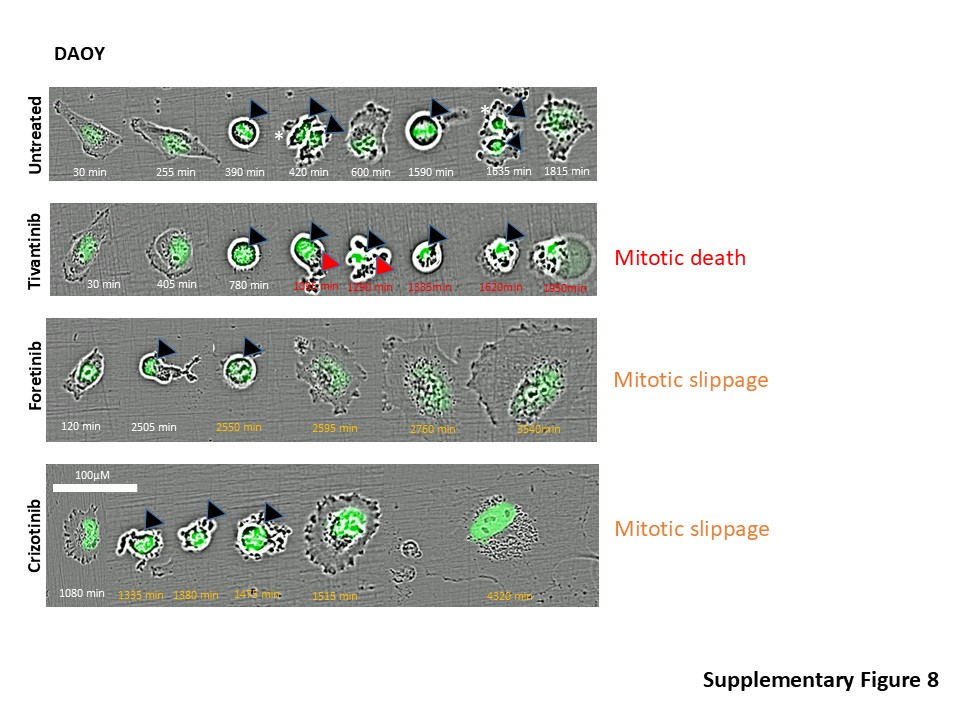

Supplement: Supplementary file 2 — Supplementary Material 2 (Supplementary Fig. 1. Gene expression analysis of tyrosine kinase receptors derived from six different publicly available datasets (Gilbertson n = 76; Hsieh n = 31; denBoer n = 51; Pfister n = 223; Delattre n = 57; Kool n = 62) compared with expression in healthy cerebellum (Roth n = 9). Heatmap representation of gene expression is based on log2 normalised gene expression signal of all analysed RTK. Only seven genes (RYK, ROR1, ROR2, EPHB2, PTK7, EPHA3, MET) out of 29 significantly modulated RTK genes (FLT3, AATYK, FGFR1, TYRO3, KIT, FGFR3, NTRK2, EPHA4, FGFR2, ROS1, RYK, EPHB3, ROR1, EPHB2, STYK1, PTK7, RON, EPHA5, ERBB3, CSF1R, EPHA3, ALK, MET, EPHA7, INSRR, RET, ROR2, PDGFRB, VEGFR3) showed an increased expression in tumour compared to normal (corrected p-value < = 0.05). Heatmap was generated by plotting gene expression values on R software. Red arrows indicate genes significantly overexpressed in MB compared to normal adult cerebella. Tumour samples comprehend a mix of MB specimens belonging to the different subgroups. Supplementary Fig. 2. (A) HGF Log2 normalised expression across six different datasets of medulloblastoma samples compared with healthy cerebella (One way ANOVA p-value = 5.53x10− 16) (Gilbertson n = 76; Hsieh n = 31; den Boer n = 51; Pfister n = 223; Delattre n = 57; Kool n = 62; Roth n = 9). Tumour samples comprehend a mix of MB specimens belonging to the different subgroups. (B) HGF Log2 normalised gene expression in Cavalli dataset (MB samples = 612) across medulloblastoma subgroups (no of samples: WNT = 70; SHH = 223; Group 3 = 144; Group 4 = 326). One way ANOVA p-value = 3.27e-26. (C) HGF Log 2 normalised gene expression across subtypes of medulloblastoma (no of samples: WNT = 21; WNT = 49; SHH = 65; SHH = 35; SHH = 76; SHH = 47; Group 3 = 67; Group 3 = 37; Group 3 = 40; Group 4 = 98; Group 4 = 109; Group 4 = 119). One way ANOVA p-value = 8.77e-34. (D) Correlation between HGF and c-MET expression (from Cavalli et a [file 12672_2026_4717_MOESM2_ESM.zip › Supplementary Figures 1 2 3 4 5 6 7 9 10 11 final/Fig. S8.JPG]

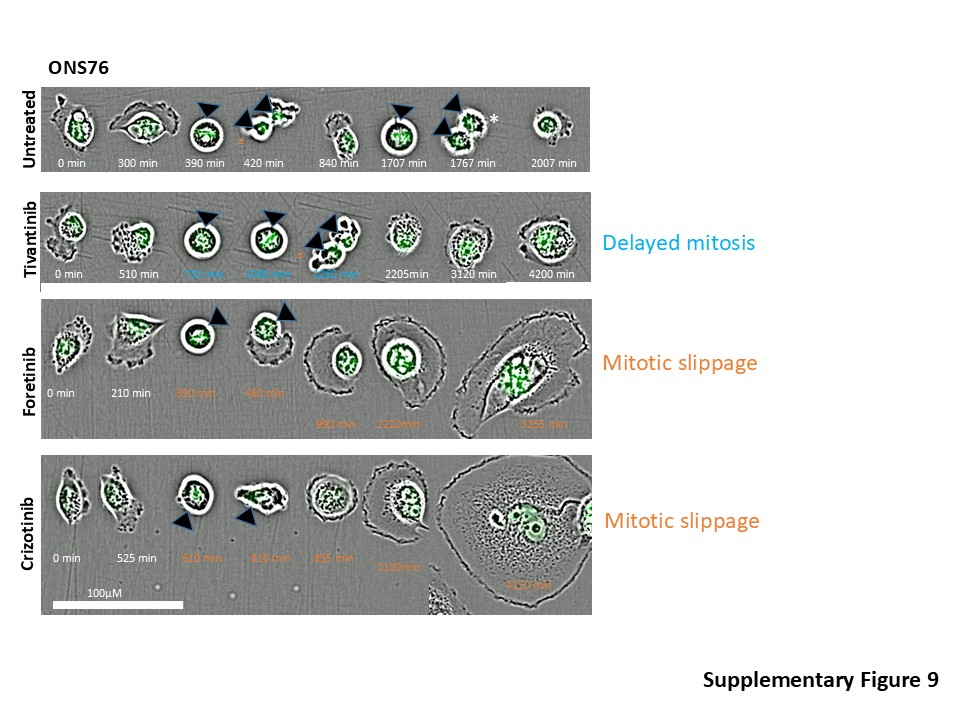

Supplement: Supplementary file 2 — Supplementary Material 2 (Supplementary Fig. 1. Gene expression analysis of tyrosine kinase receptors derived from six different publicly available datasets (Gilbertson n = 76; Hsieh n = 31; denBoer n = 51; Pfister n = 223; Delattre n = 57; Kool n = 62) compared with expression in healthy cerebellum (Roth n = 9). Heatmap representation of gene expression is based on log2 normalised gene expression signal of all analysed RTK. Only seven genes (RYK, ROR1, ROR2, EPHB2, PTK7, EPHA3, MET) out of 29 significantly modulated RTK genes (FLT3, AATYK, FGFR1, TYRO3, KIT, FGFR3, NTRK2, EPHA4, FGFR2, ROS1, RYK, EPHB3, ROR1, EPHB2, STYK1, PTK7, RON, EPHA5, ERBB3, CSF1R, EPHA3, ALK, MET, EPHA7, INSRR, RET, ROR2, PDGFRB, VEGFR3) showed an increased expression in tumour compared to normal (corrected p-value < = 0.05). Heatmap was generated by plotting gene expression values on R software. Red arrows indicate genes significantly overexpressed in MB compared to normal adult cerebella. Tumour samples comprehend a mix of MB specimens belonging to the different subgroups. Supplementary Fig. 2. (A) HGF Log2 normalised expression across six different datasets of medulloblastoma samples compared with healthy cerebella (One way ANOVA p-value = 5.53x10− 16) (Gilbertson n = 76; Hsieh n = 31; den Boer n = 51; Pfister n = 223; Delattre n = 57; Kool n = 62; Roth n = 9). Tumour samples comprehend a mix of MB specimens belonging to the different subgroups. (B) HGF Log2 normalised gene expression in Cavalli dataset (MB samples = 612) across medulloblastoma subgroups (no of samples: WNT = 70; SHH = 223; Group 3 = 144; Group 4 = 326). One way ANOVA p-value = 3.27e-26. (C) HGF Log 2 normalised gene expression across subtypes of medulloblastoma (no of samples: WNT = 21; WNT = 49; SHH = 65; SHH = 35; SHH = 76; SHH = 47; Group 3 = 67; Group 3 = 37; Group 3 = 40; Group 4 = 98; Group 4 = 109; Group 4 = 119). One way ANOVA p-value = 8.77e-34. (D) Correlation between HGF and c-MET expression (from Cavalli et a [file 12672_2026_4717_MOESM2_ESM.zip › Supplementary Figures 1 2 3 4 5 6 7 9 10 11 final/Fig. S9.JPG]
